# Endosome-microautophagy targeting chimera (eMIATAC) for targeted proteins degradation and enhance CAR-T cell anti-tumor therapy

**DOI:** 10.7150/thno.98574

**Published:** 2024-07-22

**Authors:** Kunjian Lei, Jingying Li, Zewei Tu, Chuandong Gong, Junzhe Liu, Min Luo, Wenqian Ai, Lei Wu, Yishuang Li, Zhihong Zhou, Zhihao Chen, Shigang Lv, Minhua Ye, Miaojing Wu, Xiaoyan Long, Xingen Zhu, Kai Huang

**Affiliations:** 1 Department of Neurosurgery, The Second Affiliated Hospital, Jiangxi Medical College, Nanchang University, Nanchang, Jiangxi 330006, P. R. China.; 2 Key Laboratory of Neurological Diseases in Jiangxi Province, Nanchang, Jiangxi 330031, P. R. China.; 3 JXHC Key Laboratory of Neurological Medicine, Nanchang University, Nanchang, Jiangxi, P. R. China.; 4 Department of Comprehensive Intensive Care Unit, The Second Affiliated Hospital of Nanchang University, Nanchang, Jiangxi 330006, P. R. China; 5 The MOE Basic Research and Innovation Center for the Targeted Therapeutics of Solid Tumors, Jiangxi Medical College, Nanchang University, Nanchang, P. R. China.; 6 School of Basic Medical Sciences, Nanchang University, 330031, Nanchang, P. R. China.; 7 East China Institute of Digital Medical Engineering, Shangrao, Jiangxi 334000, P. R. China.

**Keywords:** Targeted protein degradation, eMIATAC, Cancer therapy, Autophagy degradation

## Abstract

**Rationale:** Since oncogene expression products often exhibit upregulation or abnormally activated activity, developing a technique to regulate abnormal protein levels represent a viable approach for treating tumors and protein abnormality-related diseases.

**Methods:** We first screened out eMIATAC components with high targeted degradation efficiency and explored the mechanism by which eMIATAC induced target protein degradation, and verified the degradation efficiency of the target protein by protein imprinting and flow cytometry. Next, we recombined eMIATAC with some controllable elements to verify the regulatable degradation performance of the target protein. Subsequently, we constructed eMIATAC that can express targeted degradation of AKT1 and verified its effect on GBM cell development in vitro and in vivo. Finally, we concatenated eMIATAC with CAR sequences to construct CAR-T cells with low BATF protein levels and verified the changes in their anti-tumor efficacy.

**Results:** we developed a system based on the endosome-microautophagy-lysosome pathway for degrading endogenous proteins: endosome-MicroAutophagy TArgeting Chimera (eMIATAC), dependent on Vps4A instead of lysosomal-associated membrane protein 2A (LAMP2A) to bind to the chaperone Hsc70 and the protein of interest (POI). The complex was then transported to the lysosome by late endosomes, where degradation occurred similarly to microautophagy. The eMIATACs demonstrated accuracy, efficiency, reversibility, and controllability in degrading the target protein EGFP. Moreover, eMIATAC exhibited excellent performance in knocking down POI when targeting endogenous proteins in vivo and in vitro.

**Conclusions:** The eMIATACs could not only directly knock down abnormal proteins for glioma treatment but also enhance the therapeutic effect of CAR-T cell therapy for tumors by knocking down T cell exhaustion-related proteins. The newly developed eMIATAC system holds promise as a novel tool for protein knockdown strategies. By enabling direct control over endogenous protein levels, eMIATAC has the potential to revolutionize treatment for cancer and genetic diseases.

## Introduction

Several human tumors, including breast cancer, lung cancer, chronic lymphocytic leukemia and colorectal cancer 1, are closely associated with alterations in the expression of proteins in cells, emphasizing the need to develop effective methods of treating tumors by reducing or blocking the expression of carcinogenic proteins 2-5. Over the years, DNA knockout and RNA interference, which regulate protein expression levels at genome and mRNA levels, respectively, have been widely applied and undoubtedly are effective tools to change the content and function of endogenous protein expression in cells 6-8. However, both methods provide indirect regulation of proteins that may be limited in terms of speed, specificity, and reversibility 9, 10. Technologies like Clustered Regularly Interspaced Short Palindromic Repeats (CRISPR)/Cas9 can lead to off-target phenomena 11; long-lived proteins require more time to be depleted and may even be completely resistant to DNA and RNA-targeted depletion methods 12. After removing external factors, the damaged genome may be difficult to recover and cannot reverse the expression of the target protein 13. These shortcomings also limit the full applicability of these methods as research tools and clinical treatments.

The past few years have witnessed a burgeoning interest in targeted protein degradation (TPD). Within the cellular environment, protein degradation is regulated by two principal pathways: the ubiquitin-proteasome system (UPS) and the lysosomal system 14, 15. The UPS, centered on the proteasome, eliminates short-lived and misfolded proteins 14. Conversely, lysosomes degrade long-lived proteins, insoluble aggregates, and even organelles 16-18. They achieve this through various processes like endocytosis, phagocytosis, and autophagy, with higher eukaryotes having three known autophagy pathways: chaperone-mediated autophagy (CMA), macroautophagy, and microautophagy 19, 20. CMA specifically targets proteins with KFERQ-like motifs, while endosome-microautophagy involves Hsc70-mediated recognition and CMA targeting motifs (CTMs) can be found in all known proteins that serve as CMA substrates. Lysosomal-associated membrane protein 2A (LAMP2A) is combined with Hsc70 substrate, which is then transferred to the lysosome cavity for degradation 21-22. Studies have identified an endosome-microautophagy process where the transfer of cytoplasmic proteins to late endosomes (LEs) is independent of LAMP2A. However, it requires the formation of LE/multivesicular bodies (MVBs) mediated by Vps4 and Tsg101. The protein is first recognized and bound by Hsc70, which then interacts with the endosome membrane electrostatically through its cation domain. This interaction leads to membrane invagination, followed by transport of the protein to lysosomes for degradation 19, 23. These pathways have the potential to target oncogenic proteins to treat tumors.

Targeted protein degradation is emerging as a prospective approach in drug development, offering an attractive strategy for eliminating disease-related proteins 24. TPD utilizes chimeric molecules that combine a target-binding moiety with a moiety that recruits the cellular protein degradation machinery. Over the years, several types of TPD chimeras have been developed, each exploiting different degradation pathways within the cell. These include PROTACs (PROteolysis Targeting Chimeras) for ubiquitin-proteasome system targeting, LYTAC (Lysosome Targeting Chimera), abTAC (Antibody-based PROTAC), and AUTOTAC (AUTOPhagy Targeting Chimera) 25- 28. Each method offers distinct advantages. For instance, PROTACs, delivered as small-molecule polypeptides, can rapidly degrade target proteins without altering the genome 25. This approach can be effective at the cellular level, as well as in primary cells and animal models, without affecting other protein levels globally 29.

Chimeric antigen receptor (CAR) T cell therapy, which showed remarkable efficacy in treating blood diseases, holds immense potential for further development 30. However, its effectiveness in solid tumors remains limited 30, primarily due to the complex tumor microenvironment, challenges with T cell infiltration, and T cell exhaustion from sustained antigen exposure 31. Studies have revealed that targeting T cell-exhaustion-related proteins like BATF, IKZF2, and TLE4 can significantly increase the persistence and anti-tumor capabilities of CAR-T cells both *in vitro* and* in vivo* 32. In the quest to target glioblastoma (GBM), a challenging tumor encased by the blood-brain barrier, identifying better recognition targets alongside new strategies is crucial to further enhance the efficacy of CAR-T cell therapy for GBM 33.

Herein, we developed an endosome-microautophagy-targeting chimera (eMIATAC) that leverages the endosome-microautophagy-lysosomal pathway for protein degradation. eMIATAC incorporates multiple functional domains: a cell membrane permeability domain, a protein of interest (POI) binding domain, and a chaperone recognition motif, with an optional regulatory domain. Our research demonstrates that eMIATAC can target a wide range of cellular proteins for degradation with high selectivity and controllability. This versatility makes eMIATAC a promising candidate for both direct targeted therapy of tumors and for enhancing the persistence of CAR-T cells, ultimately amplifying their therapeutic efficacy. Therefore, eMIATAC holds significant potential for clinical applications in treating cancers driven by abnormally activated or overexpressed oncoproteins.

## Methods

**Materials.** Three human cell lines (HEK-293T, U251, and MDA-MB-231) were purchased from the American Type Culture Collection (ATCC). Asunaprevir (ASV), proteasome inhibitor MG132, AKT specific inhibitor MK2206, lysosomal inhibitor pepstatin A, and isopropyl β- D-1-thiogalactoside (IPTG) were obtained from MCE (USA). Macroautophagy inhibitor 3-MA, Urea, and gastrostatin were acquired from GLPBIO. Anti-Rab7A and anti-KDM5 B antibodies were purchased from Abcam. Anti-EGFR, anti-AKT (pT308), Rabbit/Mouse Normal IgG antibodies were obtained from Cell Signaling Technology (CST). Antibodies against GAPDH, GFP, Tubulin, Actin, LAMP2, Hsc70, Vps4A, Flag, His, AKT1, and AKT (pS473) were provided by Proteintech. Lysotracker was purchased from Beyotime (Shanghai, China).

**Plasmid.** To construct various plasmids, we employed several approaches. The EGFP overexpression plasmid (FV103) was a gift, and its EGFP fragment was used for further modifications. We fused a synthetic AAKFERQHMD fragment to the N-terminus of EGFP and inserted it into a pCDH-puro vector containing a myristoylated and HA-tagged Akt1, generating a transient expression plasmid (KE). Point mutations were introduced into KE using a commercial kit to create KFERA-EGFP and CTM-EGFP plasmids. Alternatively, pre-made KFERQ-GFPnanobody and vhhGFP4-KFERQ fragments were incorporated into pCDH-puro after restriction enzyme digestion. Similar strategies were used to construct plasmids expressing 2xKFERQ-2xAKTin, 2xKFERQ-cpLOV2-vhhGFP4, vhhGFP4-LOV2-2xKFERQ, His-TAT fusion proteins with 2xKFERQ or 2xAKTin, and 2xKFERQ-mHCV-NS3-EGFP or EGFP-HCV-NS3-2xKFERQ. pCDH-EF1-Luc-P2A-copGFP (Luc-GFP) was another gift. For NLS-EGFP and CAAX-EGFP plasmids, synthetic NLS or CAAX fragments were ligated to the N-terminus of EGFP in the KE vector after KFERQ motif removal. Other plasmids were constructed by combining fragments from existing plasmids with linearized vectors. To create shRNA plasmids targeting Rab7A and LAMP2, three shRNA sequences were obtained from a collaborator. Lentiviral vectors expressing these shRNAs were produced by co-transfecting HEK293T cells with shRNA plasmids along with packaging plasmids (psPAX2 and pMD2.G), kindly provided by another researcher. Lenti-CRISPR-v2-puro (v2-puro) was used for CRISPR/Cas9 knockouts. Three gRNA sequences targeting LAMP2, Hsc70, and Vps4A were designed and inserted into the v2-puro vector to generate knockout lentiviruses.

All shRNA and sgRNA targeting sequences are provided in Table S1, and all constructs were verified by DNA sequencing.

**His peptide purification.** TAT-mKV/KV and TAT-mKA/KA were cloned into the pET28-MHL vector (pET28, Addgene plasmid #26096) using standard molecular cloning techniques. The recombinant plasmid was transformed into Escherichia coli BL21 (DE3) cells and plated on LB agar plates containing 50 mg/mL kanamycin for selection. Colonies were grown overnight at 37 °C. A single colony of each transformation was picked and inoculated into an LB medium containing 50 mg/mL kanamycin. When the culture reached an OD600 of 1.2-1.4, protein expression was induced by adding isopropyl β-D-1-thiogalactoside (IPTG) at a final concentration of 1 mM. The culture was then incubated at 20 °C for 16-18 hours. Bacteria were harvested by centrifugation, and the cell pellet was resuspended in a lysis buffer. Following sonication and centrifugation, the bacterial lysate was clarified. The His-tagged polypeptides were purified using a nickel affinity chromatography column (HisTrap HP, Cytiva, 17524701) following the manufacturer's instructions. Briefly, the column was equilibrated before loading the clarified lysate. After washing the column, the bound His-tagged proteins were eluted using an elution buffer. The purity of the eluted proteins was assessed by Coomassie blue staining, and the optimal elution conditions were determined. Finally, the concentration of the purified peptides was measured by absorbance at 280 nm.

**Cell culture, transfection, and treatment.** HEK-293T and U251 cells were cultured in Dulbecco's Modified Eagle Medium (DMEM; Gibco) supplemented with 10% fetal bovine serum (FBS; Gibco), 100 U/mL penicillin, and 100 μg/mL streptomycin (Gibco). Both cell lines were maintained in a humidified incubator at 37 °C with 5% CO2. MDA-MB-231 cells were cultured in L-15 medium (Gibco) containing 10% FBS, 100 U/mL penicillin, and 100 μg/mL streptomycin in a humidified incubator at 37 °C with ambient air (100% O_2_). Cells were plated in dishes or well plates of various sizes and allowed to reach 80% confluence for transient transfection. Transfection was performed following the manufacturer's instructions using Lipofectamine 3000 (Invitrogen, L3000015). Selection drugs were added to the culture medium 6-18 hours after transfection. Cells were processed for analysis (fluorescence microscopy, flow cytometry, or protein extraction) 24-48 hours post-transfection. Lentiviral particles were concentrated using a Lentivirus concentration kit (BIOMIGA, China). Concentrated virus was added to the cell culture medium after filtration through a 0.22 μm filter membrane. Following a 12-24-hour incubation, the medium was replaced. For shRNA knockdown, cells were infected with lentivirus and screened for high gene silencing efficiency 48 hours post-infection. Selected cell lines were verified, and the culture medium was supplemented with antibiotics to maintain selection pressure. For CRISPR/Cas9 knockout, sgRNA lentivirus was used to generate single-cell clones in 48-well plates by limiting dilution in the presence of puromycin. After several weeks of clonal expansion, pure knockout cell lines were identified by Western blotting, and the line with the highest knockout efficiency was chosen for further experiments. Cells were treated with drugs or other agents when they reached 80% confluence. Typically, treatments were applied for 24 hours before analysis.

For blue-light irradiation experiments, cells were cultured in the dark or exposed to 470 nm blue light at an irradiance of 4 mW/cm^2^. Irradiated cells were used immediately for subsequent experiments.

**Western blotting and immunoprecipitation.** For WB analysis and immunoprecipitation, the following procedures were conducted as previously outlined 34. Briefly, cells were lysed using RIPA buffer (Cell Signaling Technology, USA) supplemented with protease and phosphatase inhibitors (Cell Signaling Technology, USA). Protein lysates (20 μg) were separated by SDS-PAGE using 10% gels and transferred to PVDF membranes (Millipore, USA). The membranes were then subjected to incubation with primary antibodies against the target proteins or internal references at the appropriate concentration at 4 °C, followed by incubation with horseradish peroxidase (HRP)-conjugated secondary antibodies for 2 hours at room temperature. Protein bands were visualized using a chemiluminescent imaging system (5200 Multi ChemiLuminescent, Tanon) and quantified using ImageJ software (version 1.53a, National Institutes of Health, USA).

For immunoprecipitation, cells were lysed with a Cell Lysis Buffer (Cell Signaling Technology, 9803S). Lysates were incubated with specific antibodies overnight at 4 °C on a rotary shaker. Protein A/G PLUS-Agarose beads (Santa Cruz, SC-2003) were then added and incubated for an additional 3 hours on a rotary shaker. Beads were washed five times with pre-cooled lysis buffer. Immunoprecipitated proteins were eluted with a loading buffer, boiled for 10 minutes, and subjected to WB analysis.

**Cell imaging.** Following a 24-hour transfection with the corresponding plasmids, cells were incubated with a culture medium containing lysosomal tracer (Beyotime, China) for 1 hour at 37 °C. The cells were then transferred to a 12-well plate. Coverslips pre-cooled in phosphate-buffered saline (PBS) were added to each well and soaked for three washes. Cells were subsequently fixed with 2.5% glutaraldehyde (Solarbio, China) for 15 minutes. Following three washes with PBS, the coverslips were mounted onto slides and stored in a cassette at 4 °C. Images were captured using a Leica laser confocal fluorescence microscope at 63x magnification. The colocalization of EGFP and the lysosomal marker were analyzed using ImageJ software.

**Construction of mice tumor models.** All animal experiments were approved by Animal Protection and Use Committee of Nanchang University (project approval number: NCUF-ll-2022327). Mice were randomly assigned to each experimental group. Investigators were not blinded to the allocation during the experiment. Healthy nude mice (GemPharmatech, China) were used to establish intracranial glioma models and subcutaneous BRCA models. Mice were kept in a specific pathogen-free (SPF) environment. Four-week-old nude mice were anesthetized with isoflurane. 10^5^ U251 cells were injected stereotaxically at a predetermined location (2.5 mm lateral and 1 mm anterior to the bregma). The incision was sutured, and tumor growth was monitored by recording body weight and observing general health. Mice were euthanized humanely at the endpoint, and tumor tissues were harvested for Western blotting analysis after homogenization. Six-week-old female nude mice were injected subcutaneously in the fourth pair of mammary fat pads (unilateral or bilateral) with 10^6^ MDA-MB-231 cells in a sterile environment. Tumor size (length and width) was measured periodically. Mice with established BRCA tumors and stable expression of mHK/KH-A were orally administered ASV at a dose of 10 mg/kg/day. For intracranial glioma, tumor size was monitored by bioluminescent imaging. The tumor volume was determined by the formula V = (π/6) × a^2^ × b, where 'a' and 'b' denote the short and long tumor dimensions, respectively.

**Flow cytometry.** For cells requiring EGFP intensity analysis, detachment was performed using trypsin without EDTA. The collected cells were washed and suspended in PBS. EGFP expression was detected as soon as possible using a CytoFLEX flow cytometer (Beckman Coulter, USA) at the FITC channel. At least 10,000 viable cells were obtained per sample. The flow cytometry data were analyzed with FlowJo software. (version X10.0.7r2).

**Generation of CAR-T and CAR-MT cells.** Peripheral blood mononuclear cells (PBMCs) were isolated from healthy volunteers at the Second Affiliated Hospital of Nanchang University, Nanchang, China, with informed consent. Fresh whole blood from healthy adults was used within 2 hours of collection for T-cell isolation and purification. Primary human CD3^+^ T cells were separated and purified according to the manufacturer's instructions using a human mononuclear cell separation solution (Solarbio, China) and a human T cell isolation kit (e.g., Miltenyi Biotec, Beaver). The isolated T cells were then stimulated and activated using anti-CD3/CD28 beads (e.g., Miltenyi Biotec, Beaver) at a 1:1 bead-to-cell ratio. After approximately 15 days of expansion, the T cells were separated from the beads and used for subsequent experiments or cryopreservation. For CAR transduction, the pHEFI-OptoCARCD19-mScarlet plasmid (Addgene plasmid #170465), kindly provided by John James, was used as the starting material. P2A and OPTNB sequences were inserted sequentially at the C-terminus of the CAR to generate the CAR-M construct. A three-plasmid system was used to package the CAR and CAR-M constructs into lentiviruses. Viral titers were determined, and the lentiviruses were used to infect T cells. CAR expression was identified by mScarlet fluorescence, allowing for the selection of CAR^+^ T cells.

**Cytolysis assay and Elisa assay.** The anti-tumor capability of T cells was evaluated using a luciferase-based cell lysis assay. U251-luciferase cells (1 × 10^5^ cells/well) were seeded into opaque 96-well plates (100 μl/well). Effector T cells were added at fixed or varying effector-to-target (E: T) ratios (100 μl/well), and co-cultures were incubated for the desired duration. After incubation, 10 μl of luciferin potassium salt (Macklin, China) was added to each well, followed by a 5-minute incubation at room temperature. Luminescence intensity was measured using a multimode plate reader (PerkinElmer, USA). Specific lysis was calculated as follows: Cytotoxicity (%) = (1 - [RLU of co-cultured cells]) / (RLU of target cells alone) x 100%. The concentrations of interleukin-2 (IL-2) and interferon-gamma (IFNγ) were quantified using enzyme-linked immunosorbent assay (ELISA) in accordance with the guidelines provided by the manufacturer (Thermo Fisher Scientific, USA). Supernatants (100 μl) collected from co-cultures incubated for a specific duration were used for ELISA to quantify IL-2 and IFNγ secretion.

**CCK-8 assays.** To assess the effect of mKA treatment on cell proliferation, a Cell Counting Kit-8 (CCK-8) assay (Solarbio, China) was performed according to the manufacturer's instructions. 293T, U251, and MDA-MB-231 cells were seeded in 96-well plates at a density of 2,000 cells per well in triplicates. After allowing the cells to adhere completely, 10 μL of CCK-8 solution was added to each well at six time points (0, 24, 48, 72, 96, and 120 hours). Following a 1-hour incubation, cell proliferation was assessed by measuring the absorbance at 450 nm with a microplate reader.

**Statistics and reproducibility.** The quantitative experimental results were expressed as the mean ± standard error of mean (SEM). To compare the two groups, an unpaired student's t-test was employed. The Pearson correlation coefficient was calculated to assess the colocalization of EGFP and lysosomes. Survival analysis was performed using the Kaplan-Meier model with a two-sided log-rank test. Data analysis and visualization were conducted utilizing either Microsoft Excel or GraphPad Prism 8.0.2. The representative experiments were independently replicated at least three times, yielding similar outcomes. Statistical significance was assessed at a threshold of p < 0.05. The significance levels were indicated by asterisks: *p < 0.05, **p < 0.01, ***p < 0.001, ****p < 0.0001; while "ns" denoted non-significance.

## Results

### The KFERQ motif promotes the degradation of recombinant proteins

Targeted protein degradation therapy has emerged as a promising approach for cancer treatment. Currently, around ten TPD drugs, with PROTAC molecules being the most common, are undergoing clinical trials for cancer 35. However, a major limitation of PROTACs is their restricted targeting range of E3 ligases, highlighting the need for exploring additional E3 ligases for TPD 27. Recent research on endosome-lysosome and autophagy-lysosome degradation pathways has led to the development of several novel TPD strategies targeting the lysosome, including LYTAC, AbTAC, and AUTOTAC 26-28. Nevertheless, these TPD molecules still lack the desired level of accuracy, controllability, and efficiency in degrading target proteins. To address this challenge, our study introduced a novel TPD molecule called eMIATAC. This molecule combined a lysosome-targeting motif, a regulatory domain, a transmembrane motif, and a specifically designed short peptide that binds to the protein of interest.

**Figure 1:**
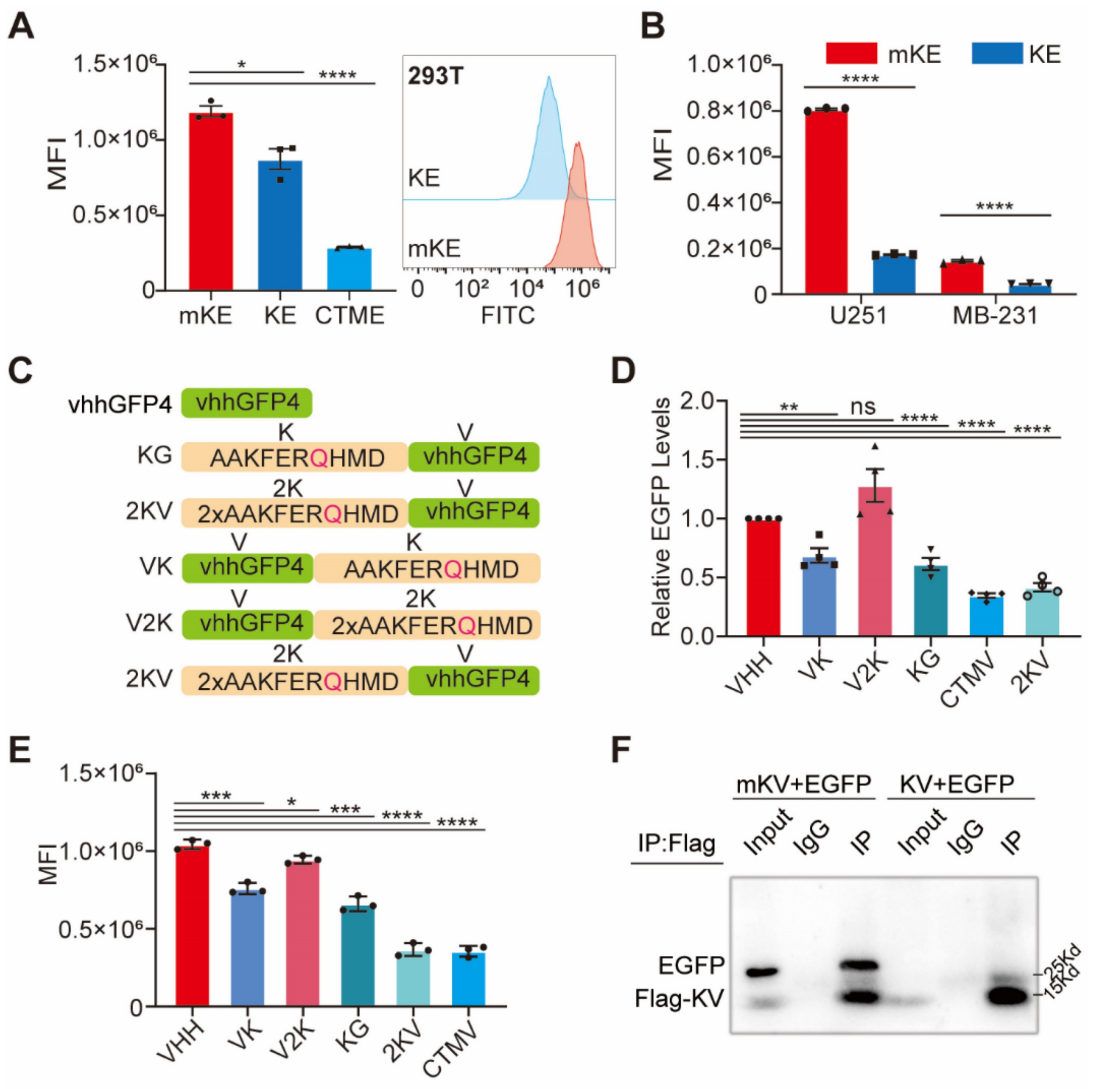
Robustness of 2×KFERQ-vhhGFP4 for EGFP degradation. (A) Left, the mean fluorescence intensity (MFI) exhibited by mKE, KE, or CTME after intracellular expression was displayed. Right, distribution of fluorescent cells in 293T cells stably expressing mKE or KE. (B) MFI of mKE and KE in U251 and MB-231 cells. (C) Schematic representation of constructs used: KG (KFERQ-vhhGFP4), 2KV (2×KFERQ-vhhGFP4), VK (vhhGFP4), V2K (control with KFERQ mutated to KV), and CTMV (positive control). (D) Western blot analysis of HEK-293T cells co-transfected with EGFP and indicated constructs. EGFP levels were significantly reduced in cells expressing 2KV and CTMV compared to KG, VK, V2K, and untransfected control (Empty). (E) MFI of intracellular EGFP in HEK-293T cells co-transfected with EGFP and indicated constructs. Data are representative of three independent experiments (mean ± SD). (F) Co-immunoprecipitation (Co-IP) assay to detect the interaction between EGFP and constructs. Western blot analysis shows strong interaction between mKV (2×KFERQ) and EGFP, while KV showed weak interaction likely due to rapid degradation of the KV-EGFP complex in 293T cells.

Chaperone-mediated autophagy is a specific protein degradation pathway containing specific pentapeptide motifs. The most commonly recognized motif is KFERQ, although others like QKILD and QRFFE have also been reported as CMA targets. This process is mediated by the chaperone protein Hsc70 36-38. Studies have shown that the KFERQ motif is relatively conserved, with the glutamine (Q) residue playing a critical role 39, 40. Additionally, exposing the KFERQ motif within a protein sequence is necessary for CMA-mediated degradation of non-CMA substrates 29. A recent study successfully designed a CMA-based degrader using the CTM motif (KFERQKILDQRFFE) 29. However, limitations remain, such as the lack of exploration for targeting membrane and nuclear proteins and the absence of certain regulatory elements. To further investigate the CMA degradation system and achieve advancements, we introduced the AAKFERQHMD sequence, containing the KFERQ motif, as a lysosome-targeting element. We constructed plasmids encoding KFERQ- Enhanced Green Fluorescent Protein (EGFP) (KE), KFERA-EGFP (Q-A mutation, designated mKE), and CTM-EGFP (CTME) (Figure S1A-B). These plasmids were overexpressed in human embryonic kidney (HEK) 293T cells. As expected, CTME exhibited significant degradation (75.7%) (Figure 1A). Interestingly, KE also displayed a robust degradation effect, confirmed by Western blotting (Figure S1C). This degradation efficiency was consistent across HEK 293T, glioma U251, and breast cancer MDA-MB-231 cells (MB-231), with KE demonstrating a slightly higher degradation rate in U251 cells (Figure S1D). To further visualize the degradation of KE, we employed fluorescence microscopy to capture the intensity of green fluorescence in the cells (Figure 1B, S1E). Notably, overexpression of KE resulted in a significant decrease in mean fluorescence intensity (MFI) compared to the mKE control group in all three cell lines (293T, U251, and MB-231) (Figure S1F-G). This suggests that KE possesses stable and reliable self-induced degradation properties.

It is now understood that EGFP fluorescence is quenched in acidic environments such as lysosomes, potentially hindering the visualization of KE accumulation within these organelles 41. Therefore, to determine the degradation pathway of the KFERQ fusion protein, we designed and overexpressed KFERQ-mRFP (KR) in U251 cells. Confocal microscopy revealed a substantial colocalization between KR and a green lysosomal tracker, suggesting KE localization to lysosomes (Figure S2A). We further investigated the role of CMA in KE degradation using specific inhibitors. MG132 (proteasome inhibitor), 3-MA (macroautophagy inhibitor), and gastrostatin-A (pepA) were employed. Notably, pepA, a lysosomal H+ ATPase inhibitor, significantly rescued KE degradation, while 3-MA and MG132 had minimal impact (Figure S3A-D). These findings collectively suggest that the N-terminal KFERQ motif in KE is stable and functional, and lysosomal activity is crucial for its degradation.

### Extended pathway of EGFP degradation: KFERQ-vhhGFP4

The discovery and application of fluorescent proteins revolutionized cell biology and biochemistry. Their advantages include ease of identification, tracking, and quantification 42. Accordingly, EGFP was extensively employed in this study. Single-chain antibodies represent another major advancement in cell biology. Camelid species possess unique heavy-chain antibodies with a single antigen-binding domain (V_H_H), also known as nanobodies. These nanobodies offer simpler selection procedures and superior stability compared to conventional antibodies 43, 44. To expand the applicability of the KFERQ motif, we introduced a nanobody targeting EGFP, known as GFPnanobody (KG) or vhhGFP4 45, 46 (Figure 1C). Recognizing that the N- or C-terminal placement of the KFERQ sequence in recombinant proteins can significantly impact its effectiveness 47, we designed additional constructs: vhhGFP4-KFERQ (VK), CTM-vhhGFP4 (CTMV), vhhGFP4 alone, 2×KFERQ-vhhGFP4 (2KV) with two KFERQ motifs at the N-terminus, and vhhGFP4-2×KFERQ (V2K) with two C-terminal KFERQ motifs (Figure 1C). These recombinant proteins were co-expressed with EGFP in 293T cells. Interestingly, 2KV displayed EGFP degradation efficiency comparable to CTMV (2KV:58.2%. CTMV:65.3%). Conversely, V2K had minimal impact on EGFP degradation, as revealed by Western blotting. Notably, KG and VK exhibited similar degradation efficiency (Figure 1D, S4A). To investigate the potentially unique role of KV in tumor cells, we co-expressed either KV or its mutated counterpart mKV (with the KFERQ motif mutated to KFERA) with EGFP in U251 and MB-231 cells, respectively. KV still demonstrated significant EGFP degradation efficiency (Figure S4B). The fluorescence images and average fluorescence intensity analysis of their expression in 293T/U251/MB-231-EGFP cells also showed that 2KV had excellent degradation efficiency on EGFP (Figure 1E, S4E-G). Immunoprecipitation analysis revealed that mKV formed a stable complex with EGFP, with a significantly higher binding affinity compared to KV (Figure 1F). Interestingly, KV displayed the ability to interact with Hsc70, which was not observed with mKV. Additionally, co-expression of KV with EGFP resulted in a significantly lower level of complex formation compared to mKV, suggesting rapid degradation of the Hsc70-KV-EGFP complex. We further detected a significant interaction between KV and Hsc70 in U251 cells, consistent with our previous observations in 293T cells. Based on these findings, we hypothesize that Flag-KV-EGFP in U251 cells is rapidly recognized by Hsc70 and targeted for lysosomal degradation. This could explain the lower amount of Flag-KV-EGFP complex captured by immunoprecipitation in U251 cells compared to 293T cells (Figure S4H).

To further investigate the role of lysosomes in KV-EGFP degradation, we employed inhibitors like MG132, 3MA, and pepA in the co-expression system containing mKV/KV and EGFP. Notably, pepA, a lysosomal H^+^ ATPase inhibitor, significantly blocked EGFP degradation. This finding suggests that lysosomes are essential for the degradation of the KV-EGFP complex (Figure S4I-L).

### The Deliverability and Universality of KV

**Figure 2:**
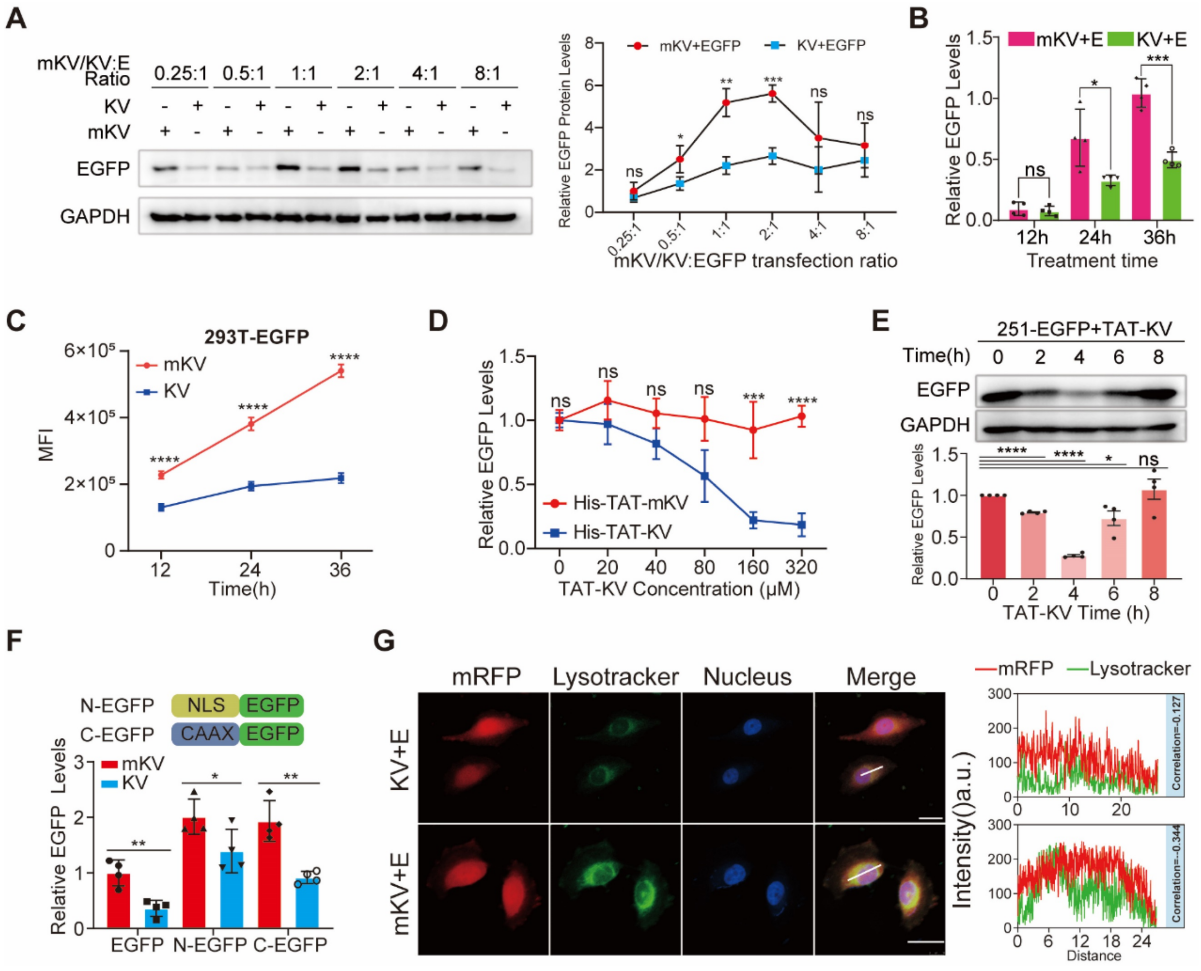
Targeted Knockdown of EGFP by KV and Its Effect on Membrane and Nuclear Proteins. (A) Western blot analysis of HEK-293T cells co-transfected with EGFP and KV at different ratios (KV: EGFP). The results suggest an optimal knockdown effect at ratios of 1:1 or 2:1. EGFP protein levels (B) and MFI (C) at different time points within cells after mKV and KV treatment in 293T cells. (D) Western blot analysis of HEK-293T cells treated with increasing doses of His-TAT-tagged KV. A dose-dependent decrease in EGFP levels is observed. (E) Western blotting showed that the efficiency of TAT-KV reached its peak at about 4h (200μM), and EGFP returned to the baseline level at about 8h, indicating that TAT-KV may maintain a short duration of action and has a good regulatory performance. (F) Confocal microscopy images of HEK-293T-YFP-mRFP cells co-expressed with mKV or KV for 24 hours. The images show the localization of mRFP (red) and lysosomes (green; stained with Lysotracker). Pearson correlation analysis revealed limitations in using this method to quantify the substantial aggregation of YFP within lysosomes. Scale bar = 25 µm. (G) Schematic representation of constructs used: NLS-EGFP (nuclear localized EGFP; NEGFP) and CAAX-EGFP (membrane-anchored EGFP; CEGFP). Co-expression with mKV or KV in 293T cells was performed. The specific knockdown effect of KV was higher on CEGFP (52.5%) compared to NEGFP (30.8%) but lower than EGFP (64.1%).

Next, we investigated the effect of different mKV/KV to EGFP co-expression ratios on EGFP levels. Indeed, the amount of transfected mKV/KV can affect the transfection reagents needed. Interestingly, at mKV/KV:EGFP ratios of 1:1 or 2:1, KV displayed a stronger degradation effect on EGFP, even though these ratios did not result in the lowest EGFP levels (Figure 2A, Figure S5A-B). Furthermore, we observed that KV-mediated EGFP degradation was time-dependent, with the difference in EGFP levels between the KV and mKV groups gradually increasing over time (Figure 2B-C, S5C-D). Having confirmed KV's remarkable ability to degrade EGFP, we explored its potential to degrade intracellular EGFP as a recombinant polypeptide that can cross the cell membrane. We achieved this by fusing a well-established membrane-penetrating peptide sequence, TAT 48, to the N-terminus of mKV/KV. These constructs were then incorporated into a bacterial expression vector for the purification of small His-TAT-KV molecules. Compared to His-TAT-mKV, His-TAT-KV effectively reduced basal EGFP expression in a dose-dependent manner. When treatment time was fixed at 4 hours, and the dose was increased from 20 μM to 320 μM, EGFP degradation efficiency reached 89% (Figure 2D, Figure S5E-I). However, 8 hours after TAT-KV treatment, EGFP knockdown gradually returned to baseline levels (Figure 2E). These findings demonstrate efficient and reversible EGFP knockdown induced by His-TAT-KV, highlighting its promising potential.

Leveraging KV's exceptional whole-cell EGFP degradation ability, we investigated its potential for degrading membrane and nuclear proteins, even though the EGFP-KV complex requires lysosomal degradation. Ras, a well-known oncogenic protein, belongs to the CAAX family of peripheral membrane proteins 49. These proteins undergo a series of C-terminal modifications (polyisoprenylation, endoproteolytic cleavage, and carboxymethylation) that render them hydrophobic and facilitate membrane association 49, 50. In cell biology, nuclear localization signals (NLS) enable recombinant proteins to accumulate in the nucleus and exert their biological functions by interacting with nuclear transport carriers 51. In the present study, we successfully inserted the membrane-targeting "CAAX" sequence (QHKLRKLNPPDESGPGCMSCKCVLS) from Ras and the common NLS sequence (KRPAATKKAGQAKKKK) into the N-terminus of EGFP, creating NLS-EGFP (NEGFP) and CAAX-EGFP (CEGFP), respectively. Co-expression of KV with NEGFP or CEGFP demonstrated KV's ability to degrade both proteins. Notably, KV displayed a slightly higher degradation efficiency for the membrane protein compared to the nuclear protein, though both were lower than for wild-type EGFP (Figure 2F, Figure S6A-E). Previous studies have shown that short synthetic peptides targeting proteins of interest can be fused with the CTM motif to direct POI degradation via the lysosome 29. Given the established interaction between Lysine-specific demethylase 5B (KDM5B) and H3K4 methylation in the nucleus 52, we designed 2×KFERQ-H3K4me0 (1-10aa) (K-H3K4me0) to target KDM5B degradation (Figure S6F). Epidermal Growth Factor Receptor (EGFR) is a common membrane protein that interacts with SH2 domain-containing proteins upon phosphorylation. We designed the 2×KFERQ-SH2 domain (K-S) to target the p-EGFR transmembrane region (Figure S6F) 53. To assess the degradation of endogenous KDM5B and EGFR, we stably expressed mK/K-H3K4me0 or mK/K-S in 293T cells. The results revealed a significant reduction in KDM5B and EGFR levels compared to the control group, with K-S exhibiting a stronger knockdown effect (Figure S6G). These findings suggest the system's potential for degrading membrane and nuclear proteins, although the underlying mechanism requires further exploration.

To elucidate whether KV undergoes degradation via the CMA pathway upon substrate binding and considering the quenching of EGFP in acidic organelles, we introduced YFP, a protein known to bind vhhGFP4 44. YFP was co-expressed with mRFP and either mKV or KV in cells for observation. Confocal microscopy was then employed to analyze the co-localization between lysosomes and YFP-mRFP in U251 cells. Interestingly, lysosomes and mRFP displayed negligible co-localization scores in both mKV and KV groups. Additionally, the reduction of mRFP was relatively consistent throughout the entire cell in the KV group, which contradicted our initial hypothesis (Figure 2G). These findings highlight the importance of investigating whether the lysosomal degradation of KV-EGFP depends on LAMP2A.

### The degradation of KV-EGFP requires Vps4A and is independent of LAMP2A

**Figure 3:**
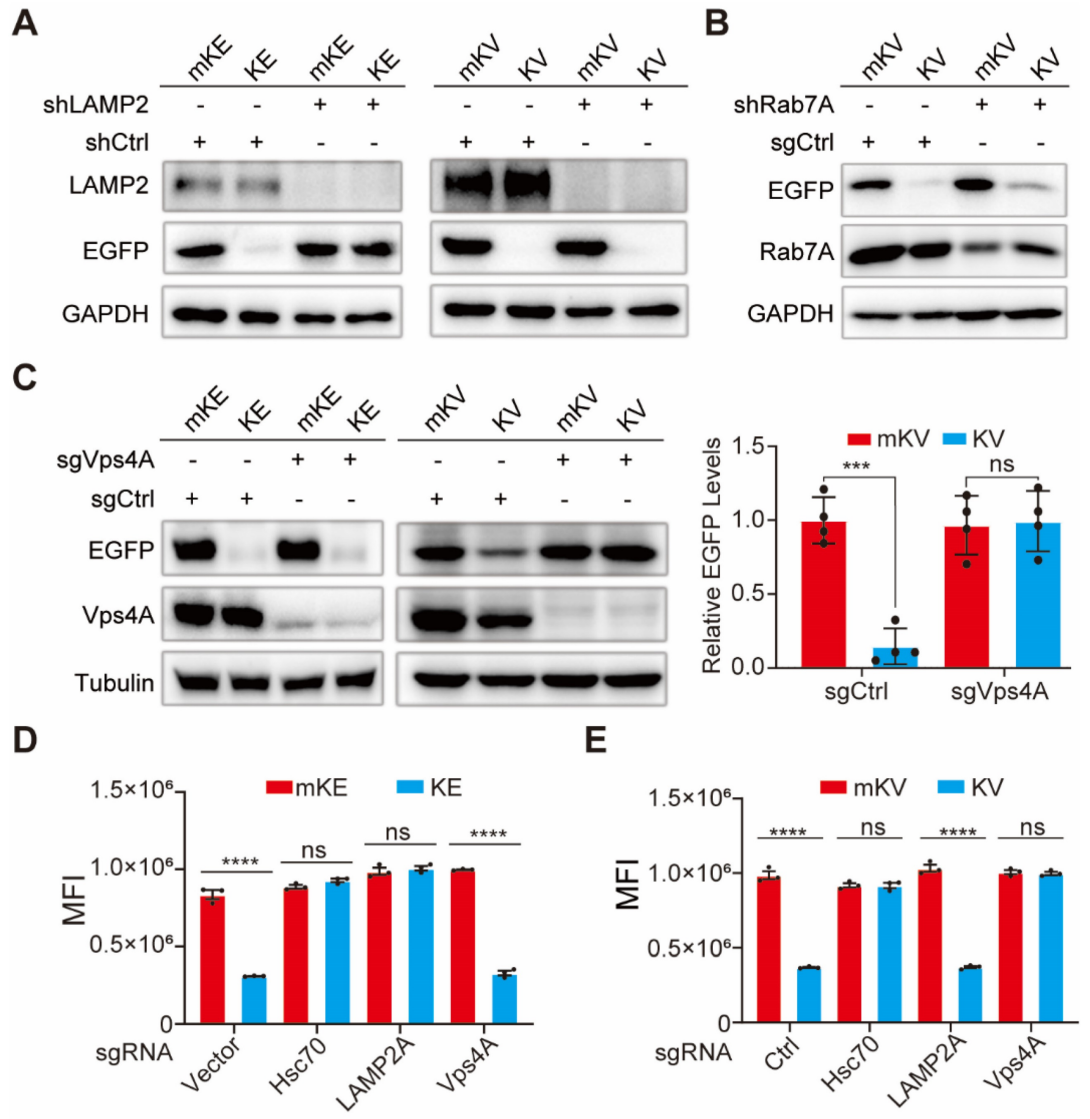
Vps4A Dependence and LAMP2A Independence in eMIATAC-Mediated EGFP Degradation. (A) Left, Intracellular protein levels of mKE or KE after LAMP2A knockout. Right, Western blot analysis of LAMP2 knockout cells treated with mKV or KV. KV retained its ability to degrade EGFP compared to the control group. (B) Western blot analysis of LAMP2A knockdown cells treated with KV. Rab7A knockdown did not affect KV-mediated EGFP degradation. (C) Left, Intracellular protein levels of mKE or KE after Vps4A knockout. Middle and Right, Western blot analysis of Vps4A knockout cells treated with KV. KV no longer significantly affected EGFP protein levels. (D) MFI of 293T-mKE or 293T-KE with knockouts of intracellular Hsc70, Vps4A, or LAMP2. (E) MFI of cells with knockouts of intracellular Hsc70, Vps4A, or LAMP2. Knockouts of Hsc70 or Vps4A, but not LAMP2, significantly impaired the function of KV.

It has been reported that following recognition and binding by Hsc70, the KFERQ-containing recombinant protein is targeted and delivered to the lysosome. During this process, the lysosomal membrane protein LAMP2A facilitates the protein's entry into the lysosome 22. To elucidate the role of LAMP2A in KV-EGFP degradation, we designed shRNA targeting LAMP2A. Intriguingly, LAMP2A knockdown inhibited KE degradation but had no significant impact on KV-EGFP degradation (Figure 3A). This finding prompted us to explore alternative degradation pathways. Hsc70 recognition of a non-CMA substrate fused with KFERQ might lead to its engulfment by the lysosome through a microautophagy-like process involving membrane fusion 54. Rab7 is a key regulator of various endosomal processes 55. Therefore, we investigated the effect of Rab7A knockdown in 293T cells. Despite achieving high Rab7A knockdown efficiency, KV-EGFP degradation remained largely unaffected (Figure 3B, S7B-C).

Since blocking vacuolar protein sorting protein 4 (Vps4) significantly reduces the lysosome's uptake of cytoplasmic proteins by hindering cargo transport 54, 56, we next focused on Vps4A. To elucidate the roles of Vps4A and Hsc70 in KV-EGFP degradation, we designed sgCtrl, sgVps4A, sgHsc70, and sgLAMP2 lentiviral plasmids using the CRISPR-Cas9 knockout system. First, we confirmed the effect of LAMP2 knockout on KE and KV-EGFP. Consistent with previous findings, KE levels were significantly restored, while KV-EGFP degradation remained unaffected (Figure S7D-E). Hsc70 and Vps4A complete knockout significantly altered 293T cell growth and was difficult to reverse. Therefore, cells with complete knockout of these genes exhibited very low survival rates. Consequently, we screened monoclonal cell clones with the highest knockout efficiency for further studies. Notably, Hsc70 knockout significantly impacted the degradation of both KE and KV-EGFP (Figure S7F-G), suggesting their degradation requires Hsc70. To investigate whether Hsc70 directs KV-EGFP to the late endosome, we co-overexpressed mKV/KV and EGFP in 293T cells with stable Vps4A knockout. Western blotting and flow cytometry analyses revealed that Vps4A knockout effectively reversed KE-EGFP degradation (Figure 3C). However, this reversal was not significant for KE degradation (Figure 3C-E, S7H).

These findings suggest that while Rab7 plays an important role in the late endosome, KV-EGFP degradation appears to be primarily dependent on Vps4A and largely independent of LAMP2. This evidence supports the hypothesis that KV-EGFP degradation occurs through LE fusion with the lysosome, resembling microautophagy. We propose the term "endosome-microautophagy targeting chimera" to describe TAT-KV, which relies on both endosomes and lysosomes for EGFP degradation. Furthermore, it is highly conceivable that KE degradation occurs through either the LAMP2-dependent CMA pathway or the endosome-microautophagy-lysosome pathway, with the latter being significantly less efficient (as evidenced by the substantial difference observed upon knockdown of fluorescence intensity). In contrast, KV-EGFP is not subject to CMA degradation but can be degraded via LE fusion with the lysosome, resulting in lower overall degradation efficiency. An alternative explanation is that KV-EGFP may interact transiently with LAMP2, but the spatial separation of KV from EGFP within the complex prevents the stable binding necessary for CMA-mediated degradation.

### Modifiable eMIATAC based on drug and blue-light switch

**Figure 4:**
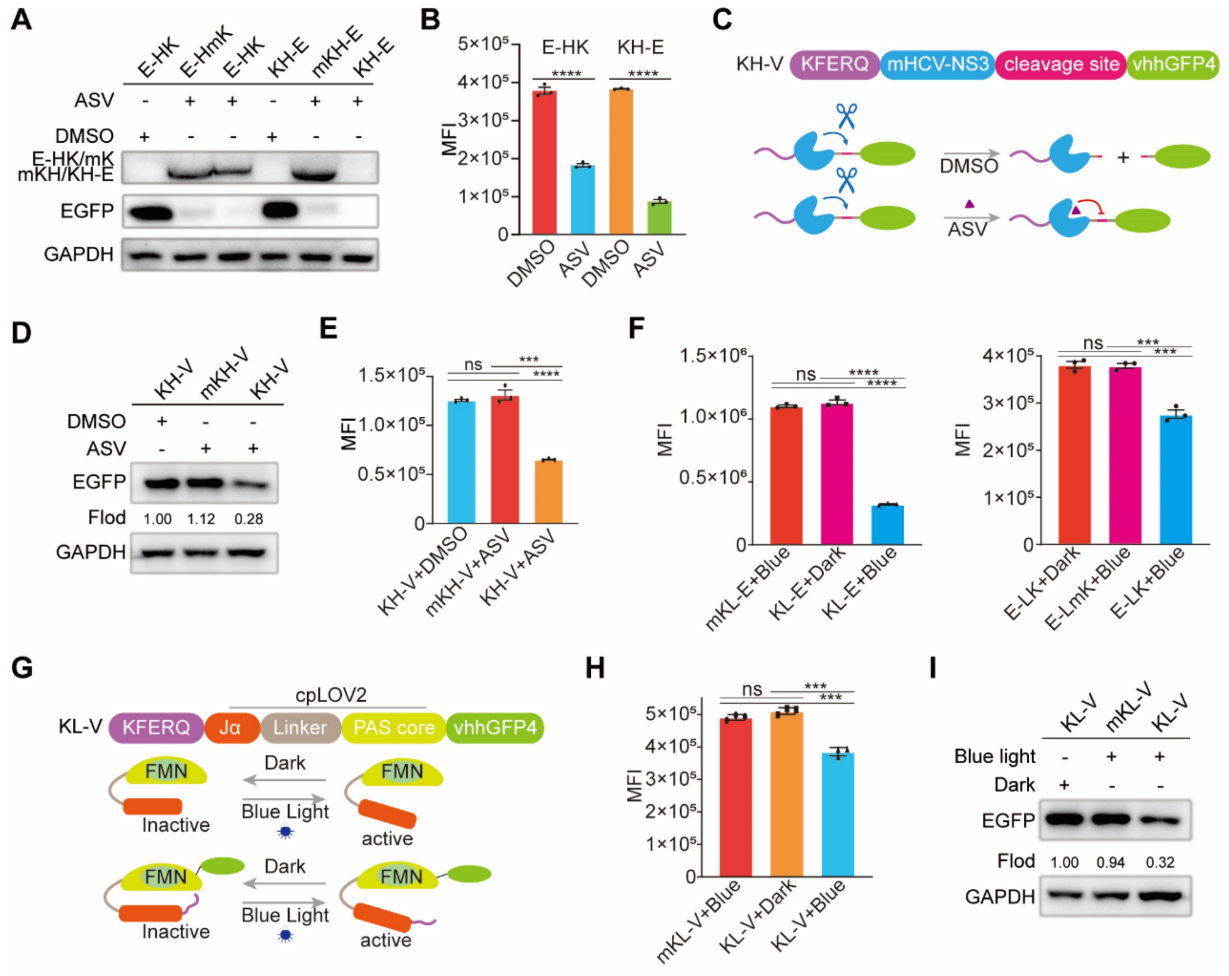
The eMIATAC containing regulatory elements can effectively regulate EGFP degradation. (A) Western blot analysis for differences in degradation efficiency of N- or C-terminus targeting KFERQ motif located at HSV-NS3/4A element. (B) MFI showed that E-HK and KH-E could degrade significantly with the ASV, and the KFERQ motif could achieve better degradation power at the N-terminal of HCV-NS3. (C) Schematic of eMIATAC containing HSV-NS3/4A under DMSO or ASV treatment. (D) Immunoblot analysis revealed that HK-V enabled EGFP degradation in the presence of ASV instead of DMSO. (E) KH-V and the action of ASV significantly regulated the degradation of EGFP. (F) The MFI of mKL-E, KL-E (Left), E-LK, and E-LmK (Right) under dark or blue light (470nm, 4mW/cm^2^) conditions. (G) Schematic of eMIATAC containing cpLOV2 under dark or blue-light treatment. (H) The regulation effect of mK/KL-V on EGFP under dark and blue light (470nm, 4mW/cm^2^) was determined by flow cytometry. (I) Immunoblotting analysis suggested that KL-V enabled EGFP degradation under blue-light (470 nm, 4 mW/cm^2^).

Having established the degradation pattern of KV-EGFP, we sought to enhance the potential applications of eMIATAC by incorporating regulatory elements. The HCV-NS3/4A protein is a multifunctional protein with a serine protease domain at its N-terminus 57. This protease domain cleaves itself unless inhibited by cell-permeable HCV-NS3/4A protease inhibitors such as danoprivir, asunaprevir (ASV), and ciluprevir 58-60. Therefore, we designed and validated recombinant sequences containing mutated HCV-NS3/4A (HCV-NS3) and KE. We investigated the difference in degradation efficiency when the KFERQ motif was placed at the N-terminus or C-terminus of the HCV-NS3 element. Based on previous studies, we tentatively constructed a group of recombinant proteins that can be regulated by ASV (Figure S8A). Consistent with prior findings, the N-terminal KFERQ motif displayed superior targeted degradation efficiency, possibly due to its full exposure (Figure 4A-B, S8B-C). The presence of ASV significantly enhanced the degradation efficiency of KFERQ-mHCV-NS3-EGFP (KH-E). Encouraged by the high-efficiency degradation of KH-E, we designed KH-vhhGFP4 (KH-V) to explore the potential for drug-controlled eMIATAC degradation, and the mechanism diagram of KH-V regulated by ASV is also drawn (Figure 4C). Interestingly, compared to the control group, KH-V significantly reduced EGFP levels in the presence of ASV compared to DMSO (Figure 4D-E, S8D-E). These results suggest that incorporating drug-control elements into eMIATAC holds promise for achieving precise and controllable degradation of proteins of interest.

It has been reported that the light-oxygen-voltage sensing domain 2 (LOV2), a subset of the Per-ARNT-Sim (PAS) family of environmental sensing domains, regulates various cellular processes 61. Exposure to intense blue light at approximately 470 nm induces conformational changes in LOV2, likely due to protonation at the adjacent N5 position. Given the small size of LOV2 and its tight N- and C-terminal proximity, we also designed a circularly permuted LOV2 (cpLOV2) variant to provide a novel cage surface while maintaining compatibility with existing LOV2-based tools (Figure S9A) 62. Consistent with previous findings, KFERQ-cpLOV2-EGFP (KL-E) exhibited superior degradation performance (Figure 4F). However, this effect was contingent upon a cell culture environment with specific light parameters: 470 nm blue light wavelength and 4 mW/cm^2^ power density (Figure S9B-C). Notably, KL-E degradation displayed time dependence, with rapid degradation observed under blue light irradiation (Figure S9D). To explore the application of blue-light regulation in eMIATAC, we designed KL-vhhGFP4 (KL-V) (Figure 4G). Importantly, KL-V co-expression with EGFP resulted in stable EGFP knockdown under specific blue light conditions (470 nm, 4 mW/cm^2^, 24 h) (Figure 4H-I, S9E). These findings demonstrate the remarkable controllability of eMIATAC, highlighting the potential for incorporating additional control elements to achieve precise POI degradation regulation under defined conditions.

### The eMIATAC knockdown AKT1 *in vitro* and *in vivo*

Our results demonstrate that KFERQ can specifically degrade KDM5B and phosphorylated EGFR (p-EGFR) when fused with short-targeting peptides. Short peptides have been established as effective tools in scientific research, acting as specific inhibitors by binding to and blocking the activity of target proteins 63. Due to the frequent hyperactivation or overexpression of AKT1 in various malignancies, anti-tumor drugs targeting AKT have become increasingly attractive. AKTin (AVTDHPDRLWAWEKF), which interacts with AKT and selectively suppresses its kinase activity, has an excellent ability to target AKT. Studies have shown that a dimerized version (2×AKTin) exhibits even stronger AKT targeting and inhibition 52, 64. In the present study, 2×KFERQ-2×AKTin (KA) recombinant proteins were designed to target the degradation of endogenous AKT (Figure S10A). Meanwhile, we designed VHL Ligand (PFSTQDTDLDLEMLAPYIPMDDDFQLR)-2×AKTin (VA) according to the basic principle of PROTAC 65, 66. After stable overexpression of both VA and KA in 293T cells, both KA and VA significantly inhibited endogenous AKT1 and pAKT to a similar level, achieving knockdown efficiencies of around 80%. However, knockdown efficiency was slightly lower in U251 and MB-231 cells, reaching 68% and 63%, respectively (Figure S10B-C). We further evaluated the effect of KA on cell proliferation, observing significantly reduced proliferation across all KA groups (Figure S10D).

**Figure 5:**
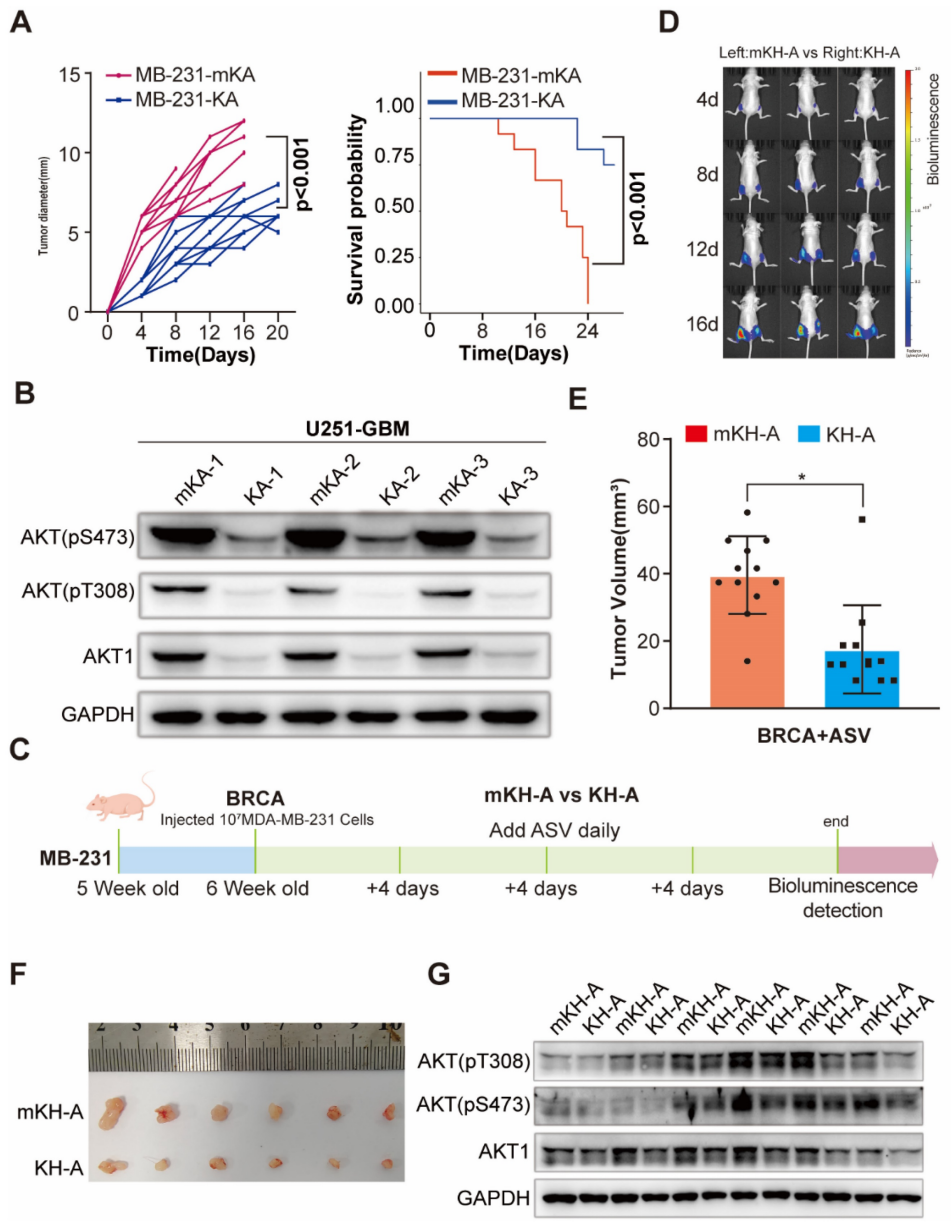
Degradation of endogenous AKT1 and pAKT by regulated eMIATAC* in vivo*. (A) Survival curve and tumor diameter comparison between mice treated with control eMIATAC (KA) and eMIATAC containing the inducible degradation domain (mKA). mKA treatment resulted in improved survival and smaller tumors. (B) Western blot analysis of AKT1 and pAKT levels in tumor tissues from nude mice xenografted with luciferase-infected U251 tumor cells. Samples were extracted from tumors expressing mKA and KA, respectively. (C) Flowchart for subcutaneous construction of the BRCA model and subsequent treatment in female nude mice (5 weeks old). (D) Bioluminescence imaging of nude mice xenografted with luciferase-infected MB-231 tumor cells. Mice received daily oral ASV after BRCA model construction. The left side shows mHK-A treatment and the right side shows HK-A treatment. (E) After separate flanking tumors were isolated, their volumes were measured (panel F). (G) Western blot analysis of lysed tumor tissues reveals that AKT1 and pAKT levels within HK-A expressing tumors were downregulated by ASV compared to controls.

To investigate the potential of eMIATAC for degrading endogenous proteins *in vivo*, we established BRCA and glioma xenograft tumor models using MB-231 and U253 cells stably expressing KA and mKA (Figure S10E). After several weeks under similar conditions, mice overexpressing KA in both glioma and BRCA models exhibited significantly improved survival rates compared to other groups. Additionally, these mice had smaller tumor volumes or bioluminescence intensity. Western blot analysis of extracted tumor tissues confirmed lower AKT and pAKT levels in KA mice compared to mKA mice (Figure 5A-B, S10F-H), further supporting the improved prognosis.

To further investigate eMIATAC's ability to degrade endogenous AKT, purified His-mKA/KA protein was added to the culture medium of 293T cells at a concentration of 200 μM for 4 hours. This treatment significantly reduced both AKT and pAKT levels, unlike MK2206, which only downregulated pAKT (Figure S11A). Finally, we explored the therapeutic potential of eMIATAC, using previously validated drugs to regulate its activity in a BRCA xenograft animal model. We designed and verified the *in vitro* efficacy of mKH-2xAKTin (mHK-A), KH-2xAKTin (KH-A), mKL-2xAKTin (mKL-A), and KL-2xAKTin (KL-A) (Figure S11B-E). Nude mice were subcutaneously injected with MB-231 BRCA cells stably expressing either mKH-A or KH-A in their inguinal regions. After four weeks, mice were regularly administered ASV orally while tumor growth was monitored in real-time. Bioluminescence imaging revealed significantly limited tumor growth on the side overexpressing KH-A compared to the control side. Western blot analysis confirmed that AKT and pAKT levels in BRCA tumors on the HK-A side were maintained at low levels (Figure 5C-G, S11F-H). These findings suggest that eMIATAC can be utilized to influence tumor development and progression under drug control.

### Enhancing the tumor-killing effect of CAR-T cells through targeted degradation of exhaustion-related proteins using eMIATAC

**Figure 6:**
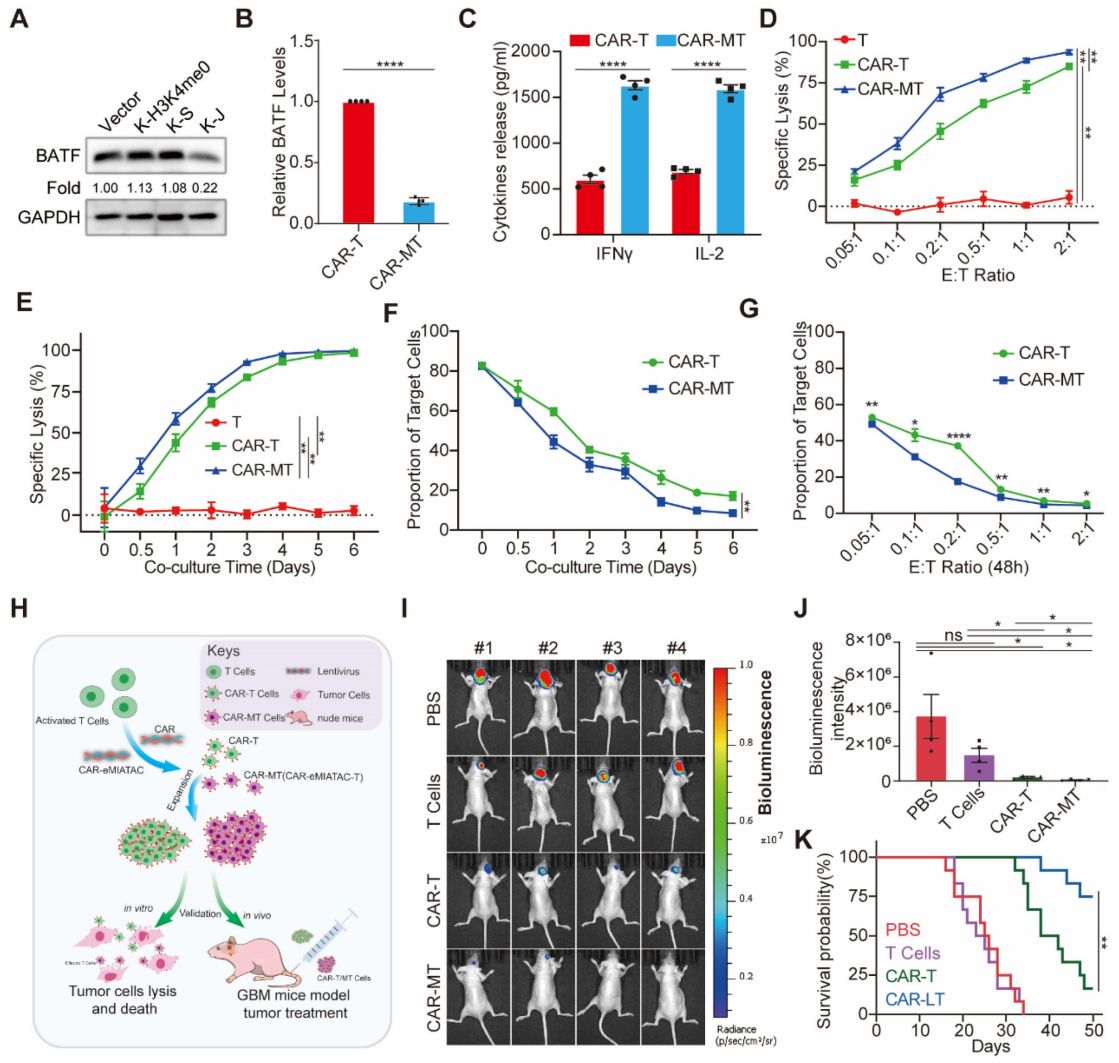
The eMIATAC was used to inhibit T cell exhaustion and enhance its tumor cell killing function. (A) Overexpression of different eMIATACs into primary T cells, only KFERQ-JUN binding domain (K-S) targeting BATF can effectively reduce the protein level of BATF. (B) Effector cytokine production of primary T cells after introduction to CAR or CAR-M (n = 4). (C) The protein level of BATF in primary T cells after the introduction of CAR or CAR-M (n = 4). (D) Cytotoxic activity of CAR-T or CAR-MT against U251-luciferase at different E:T ratios (n = 3). (E) Cytotoxic activity of CAR-T or CAR-MT against U251-luciferase at different time points (n = 3). (F) The remaining proportion of U251 cells at different time points after co culturing CAR-T or CAR-MT with U251 EGFP cells at a ratio of 0.2:1 (E: T) (n = 3). (G) The remaining proportion of U251 cells after co-culturing CAR-T or CAR-MT with U251 EGFP cells at different E:T ratios (n = 3). (H) Schematic diagram of CAR-MT validation *in vitro* and* in vivo*. Intracranial bioluminescence images (I) and differences in bioluminescence intensity (J) captured on the 28th day after T cell injection in nude mice. (K) Monitoring the survival status of mice after T cell injection.

To improve the anti-tumor efficacy of CAR-T cells using eMIATAC, we initially focused on targeting BATF, a protein associated with T-cell exhaustion 32. Previous data indicated that JUNB contains a domain (amino acids 259-332) that interacts with BATF 67. Based on this finding, we designed the 2×KFERQ-JUNB binding domain (K-J) sequence to target BATF degradation in primary T cells. This eMIATAC recombinant was then integrated into the C-terminus of the CAR sequence, creating CAR-M. First, we observed that the purity of isolated CD3^+^ T cells was significantly higher compared to PBMCs (Figure S12A). Following activation and expansion, we transduced T cells with Vector, K-H3K4me0, K-S, and K-J constructs. As expected, only K-J efficiently reduced BATF protein levels in T cells (Figure 6A). Subsequently, CAR or CAR-M was introduced into the T cells (generating CAR-T and CAR-MT cells, respectively). Notably, CAR-MT cells maintained low BATF protein levels, while BATF mRNA levels and CAR protein levels remained largely unaffected (Figure 6B, S12B-C). Importantly, CAR-MT cells displayed significantly higher levels of IL-2 and IFNγ, two key T-cell activation markers 30, suggesting a heightened activation state and potentially slower exhaustion compared to CAR-T cells.

To further evaluate the* in vitro* tumor cell killing efficacy of CAR-MT cells, we engineered U251 cells to stably express CD19-EGFP-P2A-luciferase. We then employed a luciferase-based cytotoxicity assay. Both CAR-T and CAR-MT cells demonstrated substantial killing activity against U251 cells, achieving over 60% cytotoxicity at a 0.2:1 effector-to-target (E: T) ratio (CAR-MT cells: U251 cells) after one day of co-culture (Figure 6D). Notably, at a fixed E:T ratio of 0.2:1, CAR-MT cells exhibited significantly enhanced killing activity compared to CAR-T cells at various time points (Figure 6E). We further monitored the viability of target cells during co-culture. Consistently, CAR-MT cells displayed faster and more efficient target cell lysis compared to CAR-T cells (Figure 6F-G, S12D, S13A).

To assess the therapeutic efficacy of CAR-MT cells *in vivo*, we established an orthotopic glioma model in nude mice using the previously described U251 cells (Figure 6H). The detailed procedures of the *in vivo* experiment are provided in Figure S13B. Briefly, mice were divided into four groups (PBS, T cells, CAR-T cells, and CAR-MT cells) with 12 mice per group. Starting three days after tumor formation, mice received either PBS or an intravenous injection of 10^6^ T/CAR-T/CAR-MT cells. A booster injection was administered on the tenth day. Live imaging performed on day 28 post-tumor formation revealed that CAR-MT cells exhibited significantly superior killing effects on the glioma compared to CAR-T cells (Figure 6I-J). The survival curve further demonstrated that CAR-MT cell therapy significantly improved the prognosis of glioma-bearing mice (Figure 6K, S13C).

Overall, these data provide compelling theoretical and empirical evidence for the feasibility of *in vivo* protein knockdown strategies using eMIATAC. eMIATAC offers a promising therapeutic approach by directly degrading abnormal proteins in tumor cells while also enhancing the persistence of CAR-T cells, ultimately improving the efficacy of tumor treatment.

## Discussion

While mature CRISPR gene knockout technology and RNA interference remain the predominant methods for analyzing protein function, they have limitations. These techniques primarily achieve target protein knockdown by indirectly blocking its expression. This approach can lead to a delay in achieving the desired knockdown or knockout effect. Additionally, a risk of unpredictable activation of cellular compensatory mechanisms and accumulation of defects exists 12.

EGFP offers several advantages for protein research: (1) it is readily detectable by flow cytometry, (2) it enables semi-quantitative analysis through Western blotting, (3) its fluorescence allows for rapid visualization of location and abundance using fluorescence or confocal microscopes, (4) it has minimal impact on cellular physiology and biochemistry, and (5) in most cases, fusing EGFP to a protein doesn't alter its activity 68-70. However, it should be noted that EGFP fluorescence is quenched in acidic environments like lysosomes 45. In the present study, we initially employed EGFP for preliminary research and development of the eMIATAC system. Subsequently, we successfully utilized eMIATAC to degrade DRR2 and KDM5B with high efficacy. Finally, we targeted AKT, a protein frequently overexpressed or overactivated in cancer, for further validation, achieving promising results.

Herein, we first designed and tested the KE degron sequence. Mutation of the KFERQ motif to KFERA abolished recognition by Hsc70, leading to lysosomal targeting and degradation of the recombinant protein. This finding highlights the critical role of the glutamine (Q) residue within the KFERQ-like motif. Additionally, we observed that placing KFERQ at the N-terminus of the recombinant protein resulted in higher knockdown efficiency. Furthermore, the deletion of Hsc70 or Vps4A significantly impaired the knockdown efficacy of eMIATAC. These results underscore the importance of both the degron sequence and proper utilization of regulatory elements to achieve efficient and targeted protein degradation.

For optimal performance, eMIATAC requires several key features: a KFERQ-like motif positioned at the N-terminus, selection of polypeptides with high target protein binding ability and specificity, incorporation of appropriate regulatory elements, and the presence of endosome-lysosomal machinery like Hsc70 and Vps4A. This combination enables eMIATAC to achieve high accuracy, efficiency, and controllability in protein degradation of interest. Similar to CMA-based degraders, eMIATAC utilizes the molecular chaperone Hsc70. Ultimately, POI is directed to lysosomes for degradation. Additionally, membrane-penetrating sequences can be used to facilitate cellular entry. However, eMIATAC possesses distinct advantages. Upon Hsc70 recognition of the KFERQ-like motif, the Hsc70-eMIATAC-POI complex interacts with the late endosome rather than LAMP2A, ultimately leading to lysosomal degradation via membrane fusion. In contrast, the targeted LAMP2 complex cannot directly deliver POI to lysosomes. This phenomenon was confirmed using LAMP2 knockout cells, confocal microscopy, Western blotting, and flow cytometry. We hypothesize that eMIATAC smoothly enters lysosomes upon interaction with LAMP2A, while POI separates from eMIATAC during an allosteric process, preventing its capture by the lysosome. This hypothesis is supported by the efficient degradation of KE via the CMA pathway.

Most importantly, this study presents an improved method for *in vitro* and *in vivo* protein knockdown. Unlike traditional methods that indirectly regulate gene expression at the DNA or RNA level, eMIATAC directly targets and guides most, but not all, cellular proteins to lysosomes for degradation. We demonstrate that eMIATAC offers a stable, versatile, efficient, and controllable protein degradation system. In HEK293T cells, eMIATAC effectively degrades cytoplasmic, nuclear, and membrane proteins. While targeting proteins in other cellular compartments remains a future goal, the potential target range for proteins of interest remains extensive. Emphasis was placed on improving eMIATAC to degrade proteins in new subcellular locations. Interestingly, our study found that eMIATAC exhibits higher degradation efficiency for membrane proteins compared to nuclear proteins, contrasting with LYTAC's strength in degrading membrane proteins. This difference may be explained by the partial recognition of KFERQ by Hsc70 after POI binds to the eMIATAC binding domain. This partial recognition might induce membrane invagination, facilitating the movement of the complex to the late endosome and ultimately leading to lysosomal degradation of the POI. Alternatively, for some nuclear and membrane proteins, eMIATAC binding might occur immediately in the cytoplasm after protein folding and processing, ultimately directing the targeted POI to lysosomes for degradation.

Our findings demonstrate several key advantages of eMIATAC compared to traditional knockdown systems that target DNA or RNA. Firstly, the purified, small-molecule polypeptide component of eMIATAC can rapidly reach and degrade the target protein. This small molecule only requires the addition of a specific positioning signal based on the target's location within the cell. Importantly, the degradation effect automatically ceases after eMIATAC is metabolized. Secondly, eMIATAC allows for the controlled knockdown of the target protein in a time- and concentration-dependent manner. This system exhibits high specificity for the POI and minimizes off-target effects on other proteins. This translates to the specific knockdown of the desired target. Notably, the addition of regulatory elements allows for eMIATAC to be delivered at the gene level. Subsequent control of these regulatory elements (through drug administration, physical, or chemical methods) can then activate the degradation of POI. This approach offers a unique advantage in terms of controlling protein degradation. In our study, eMIATAC not only directly targets endogenous proteins in tumor cells but also demonstrates a novel application by targeting exhaustion-related proteins within T cells. This approach successfully enhances T cell stability, maintains sustained activation levels of CAR-T cells, and ultimately improves the efficacy of tumor treatment. These findings suggest that eMIATAC has the potential to provide a new therapeutic strategy for current solid tumor immunotherapies.

Although the delivery and stability of polypeptides as therapeutic drugs pose challenges, the development of the latest modification technology, such as reverse transcription polypeptides, has significantly improved the delivery and pharmacological characteristics of polypeptides 71. In the field of protein interaction, the functional domains of numerous proteins that can be stably bound have not yet been identified, which means that the POIs for eMIATAC are still limited 72. In addition, reliable regulatory elements are rare, although research on allosteric proteins has gradually increased in recent years 61. However, with the development of science and technology, the incorporation of various theories will give eMIATAC a wider range of applications.

Our study revealed a novel mechanism for eMIATAC-mediated protein degradation. We observed that upon recognition by a chaperone, the CMA-targeting motif can be delivered to lysosomes for degradation through two distinct pathways. One pathway involves direct entry via the LAMP2-dependent CMA pathway. Alternatively, eMIATAC can be directed to the late endosome in a Vps4A-dependent manner, where cargo is ultimately delivered to lysosomes through membrane fusion. A comprehensive illustration of the CMA and endosome-microautophagy-lysosome pathways involved is presented in Figure S13. These findings demonstrate the versatility of eMIATAC in targeting proteins for degradation. Our *in vitro* and *in vivo* validation studies highlight the potential of eMIATAC to target oncoproteins and other disease-related proteins, suggesting its promise as a novel therapeutic strategy with significant clinical application prospects.

## Supplementary Material

Supplementary figures and table.
