# Supplementary material for: Endosome-microautophagy targeting chimera (eMIATAC) for targeted proteins degradation and enhance CAR-T cell anti-tumor therapy
Source: Theranostics. 2024 Jul 22;14(11):4481–98. doi: 10.7150/thno.98574 (PMC11303074; doi:10.7150/thno.98574)
Supplement: Supplementary file 1 — Supplementary figures and table. [file thnov14p4481s1.pdf]

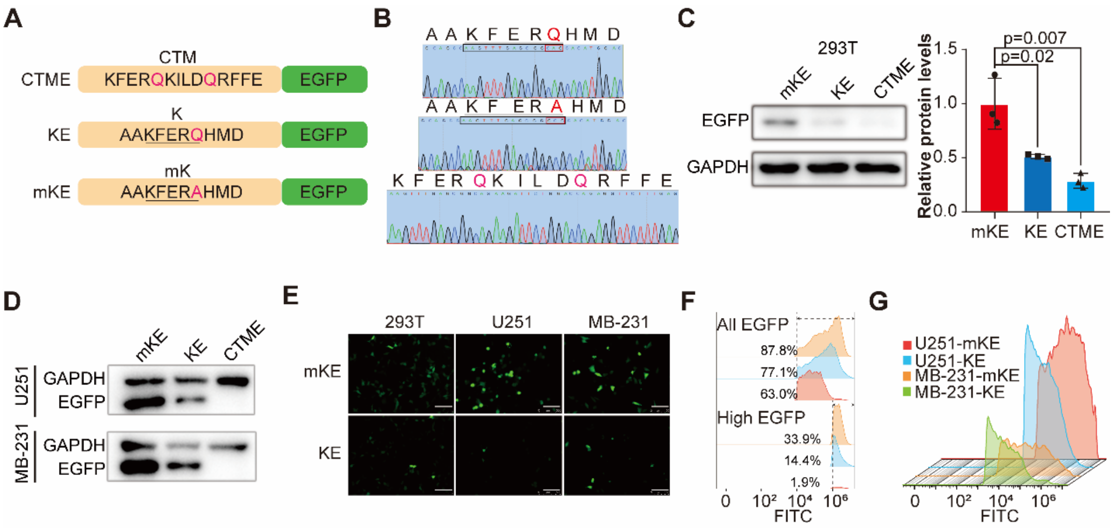

Figure S1: Degradation of KFERQ motif fusion protein by CMA pathway. (A) Schematic diagram of KFERQ-EGFP (KE), KFERA-EGFP (mKE), and CMA Targeting Motif-EGFP (CTME). (B) Sequencing of KFERQ motif of KE and CTME sequences and mutation sites of mKE. (C) Immunoblotting of cell lysates to detect indicated proteins. HEK-293T cells were used to express mKE, KE, and CTME separately with an overexpression system to quantitatively analyze the content of EGFP, and GPADH was used as the loading control. Compared with the mKE group, EGFP in the KE group and CTME group decreased by 50.8% and 70.9% respectively. (D) Immunoblot showed that the level of KE in U251 and MB-231 tumor cells were lower than mKE. And (E) displayed the distribution and intensity difference of EGFP in different groups captured by fluorescence microscope. The scale bar indicates 100 μm. (F) Flow cytometry was utilized to detect the green fluorescence intensity of mKE, KE, and CTME after 12 hours of expression based on the FITC channel. And (G) showed a 293T FITC channel with stable expression of mKE and KE.

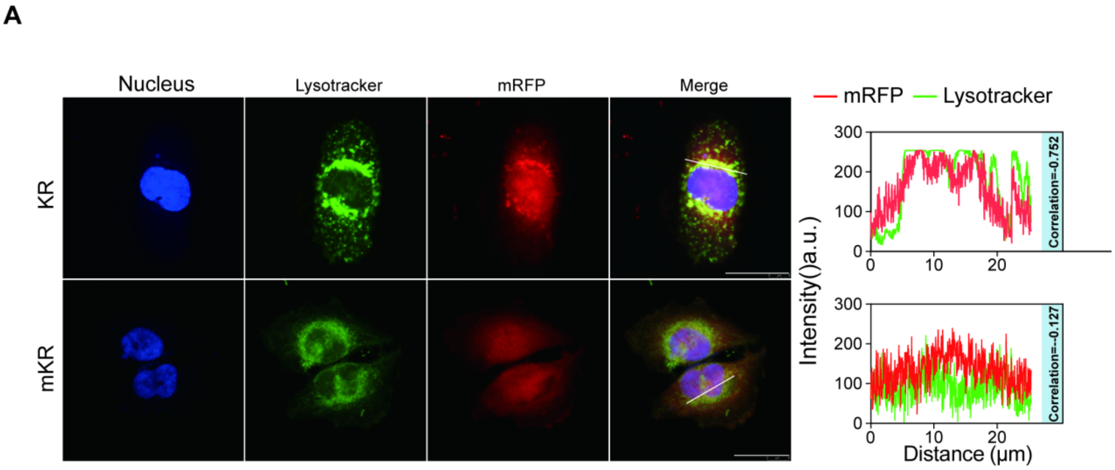

Figure S2: Validation of KFERQ-mRFP (KR) protein degradation in U251 cells. (A) After transfection of U251 cells with KFERA-mRFP (mKR) and KR for 24h, the captured confocal images showed the localization of lysosome (green lyso-Tracker) and

mRFP, and Pearson correlation analysis showed the co-localization correlation score of lysosomes and mRFP in the cells of mKR and KR groups. KR aggregates in lysosomes remarkedly, while mKR was almost uniformly distributed in the whole cell space without significant aggregation. The scale bar indicates 25 μm.

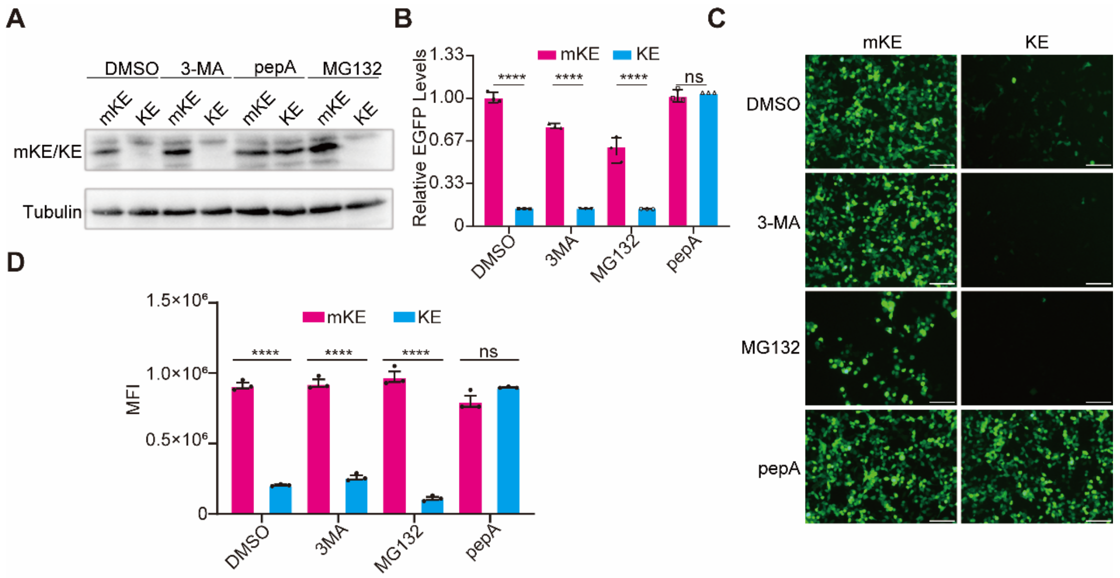

Figure S3: KE was degraded via the CMA pathway. (A) After individual transfections with mKE and KE in 293T cells, Treatment with or without macroautophagy inhibitor 3-methyladenine (3MA; 5 mM), proteasome inhibitor (MG132; 10 μM), or pepstatin A (PepA; 10 μM) for 24h. Immunoblotting revealed that Pep A significantly rescued KE degradation and (B) showed a quantitative analysis of the EGFP levels of mKE and KE, and no significant difference was observed between mKE and KE after pep A treatment. After mKE and KE were treated with multiple drugs, EGFP was captured by fluorescence microscopy (C) and examined by flow cytometry (D). The scale bar indicates 100 μm.

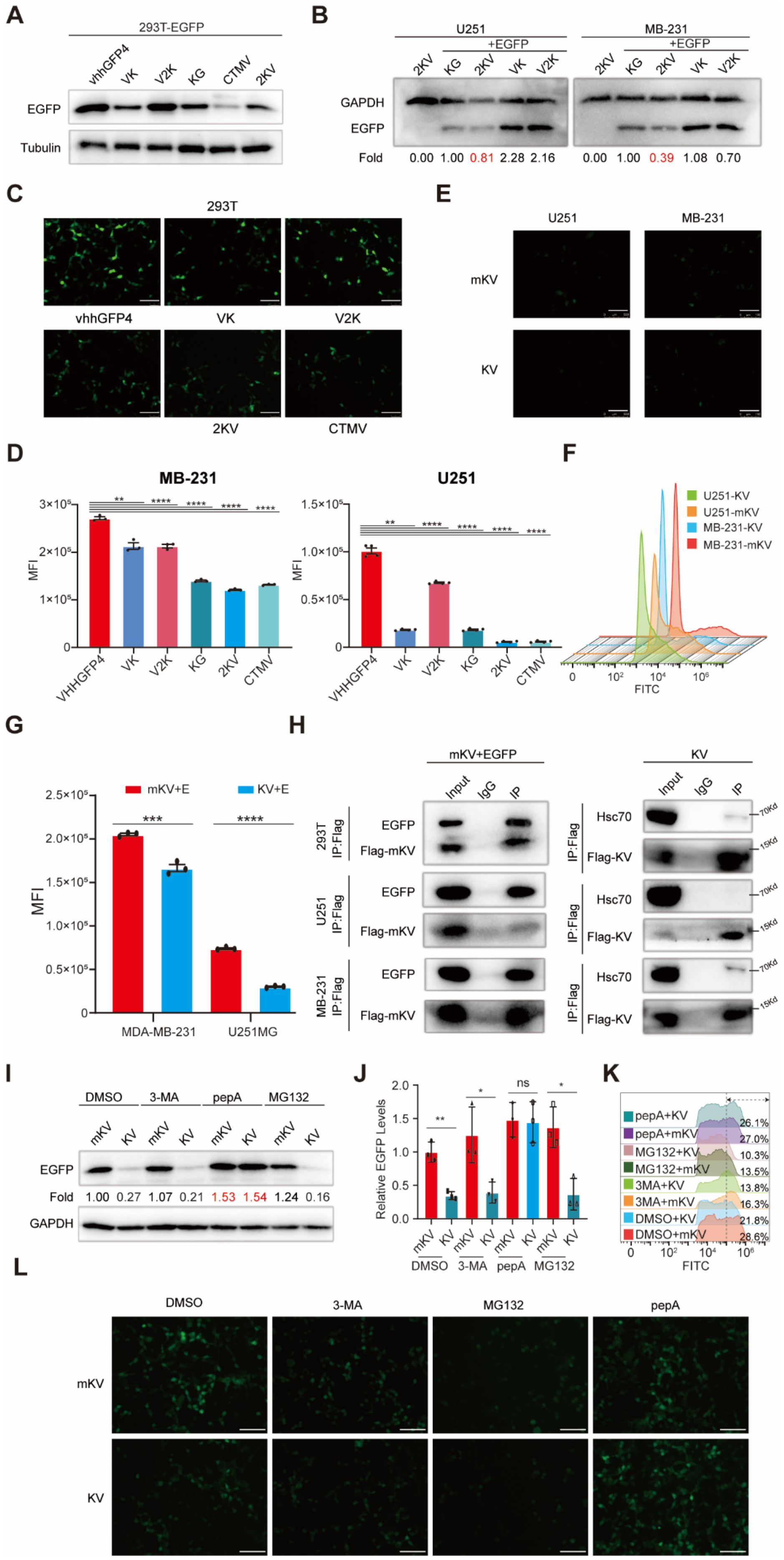
A
293T-EGFP
vhhGFP4
VK
V2K
KG
CTMV
2KV
EGFP
Tubulin
B
U251
MB-231
+EGFP
GAPDH
EGFP
Fold
0.00 1.00 0.81 2.28 2.16
0.00 1.00 0.39 1.08 0.70
C
293T
E
U251
MB-231
mKV
KV
D
MB-231
U251
MFI
VHHGFP4
F
U251-KV
U251-mKV
MB-231-KV
MB-231-mKV
FITC
G
mKV+E
KV+E
MDA-MB-231
U251MG
H
mKV+EGFP
Input
IgG
IP
293T IP:Flag
U251 IP:Flag
MB-231 IP:Flag
Flag-mKV
Hsc70
Flag-KV
IP:Flag
I
DMSO
3-MA
pepA
MG132
Fold
1.00 0.27 1.07 0.21 1.53 1.54 1.24 0.16
GAPDH
J
Relative EGFP Levels
ns
K
pepA+KV
pepA+mKV
MG132+KV
MG132+mKV
3MA+KV
3MA+mKV
DMSO+KV
DMSO+mKV
26.1%
27.0%
10.3%
13.5%
13.8%
16.3%
21.8%
28.6%
L

Figure S4: Validation of intracellular targeted knockdown of EGFP by the KV system. (A) In HEK-293T cells, EGFP was co-expressed with V, KG, 2KV VK, V2K, and CTMV respectively. Flow cytometry suggested that 2KV and CTMV could significantly knockdown the level of EGFP. (B) In U251 and MB-231 cells, after transfection with 2KV alone, EGFP co-expressed with KG, 2KV, VK, and V2K, respectively, the levels of EGFP were determined by immunoblot, with Tubulin as a loading control. Fluorescence microscopy (C, E) and flow cytometry (D, F, G) were used to detect and analyze EGFP intensity in 293T, U251, and MB-231 cells. (D) Transient transfection (Left) or stable expression (Right) of V, KG, 2KV VK, V2K, and the average fluorescence intensity 24 hours after EGFP was introduced into the cell. (H) Co-immunoprecipitation revealed that intracellular mKV interacted significantly with EGFP in 293T, U251, and MB-231 cells, whereas KV was detected to bind Hsc70 reciprocally. Applying a co-expression system to co-transfect EGFP with mKV or KV separately within 293T cells, immunoblotting (I, J), flow cytometry (K), and fluorescence microscopy (L) were used to examine the levels of EGFP after DMSO, 3-mA, MG132 and PEP a treatment from different principles. The scale bar indicates 100 μm.

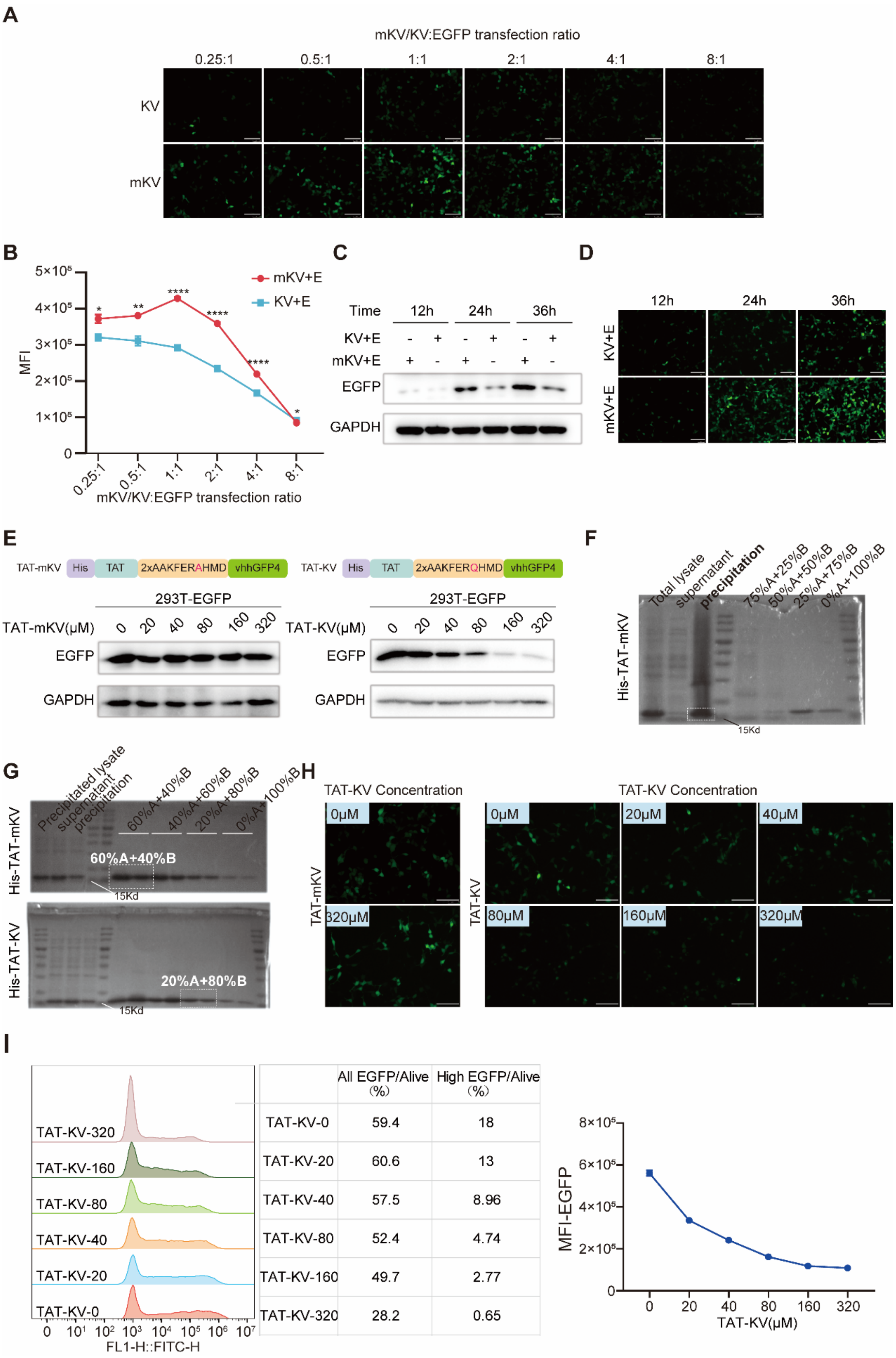

Figure S5: Characteristics of KV target knockdown EGFP, efficient validation of His-TAT-KV, and micro-distribution of KV-EGFP inside U251 and MB-231 cells. Captured photos (A), and MFI (B) both revealed that KV could have a preeminent EGFP-specific

knockdown effect (more significant difference) when KV: EGFP transfection ratio was 1:1 or 2:1. 293T cells were co-transfected with EGFP together with mKV or KV, and the fluorescence intensity and distribution of the cells were analyzed using immunoblotting (C) and fluorescence microscopy (D) at 12 h, 24 h, and 36 h, respectively, which showed that KV can stably deplete EGFP, whereas mKV cannot prevent the sustained accumulation of EGFP. (E) Left, schematic representation of His-TAT-mKV and His-TAT-KV, and bottom showed that 293T cells were given different concentrations of His-TAT-KV or His-TAT-KV, and the results of quantitative analysis of immunoblotting showed that the degradation of EGFP presented a concentration dependence of TAT-KV, and the degradation efficiency of EGFP reached 81.4% at 320 μM. Right, His-TAT-mKV was purified by nickel column purification of His fusion protein. (F) Coomassie blue staining showed that only a small amount of His-TAT-mKV was contained in the supernatant of bacterial lysis. (G) Coomas blue staining showed that His-TAT-mKV or His-TAT-KV mainly existed in the cracking supernatant of bacterial lysis precipitation. To obtain the target product with higher purity, the elution conditions in the white dashed box were selected in the experiment. (H) displayed the fluorescent images of EGFP in response to different concentrations of TAT-KV. And (J) showed the results of the flow cytometry after EGFP was treated with TAT-KV. The scale bar indicates 100 μm.

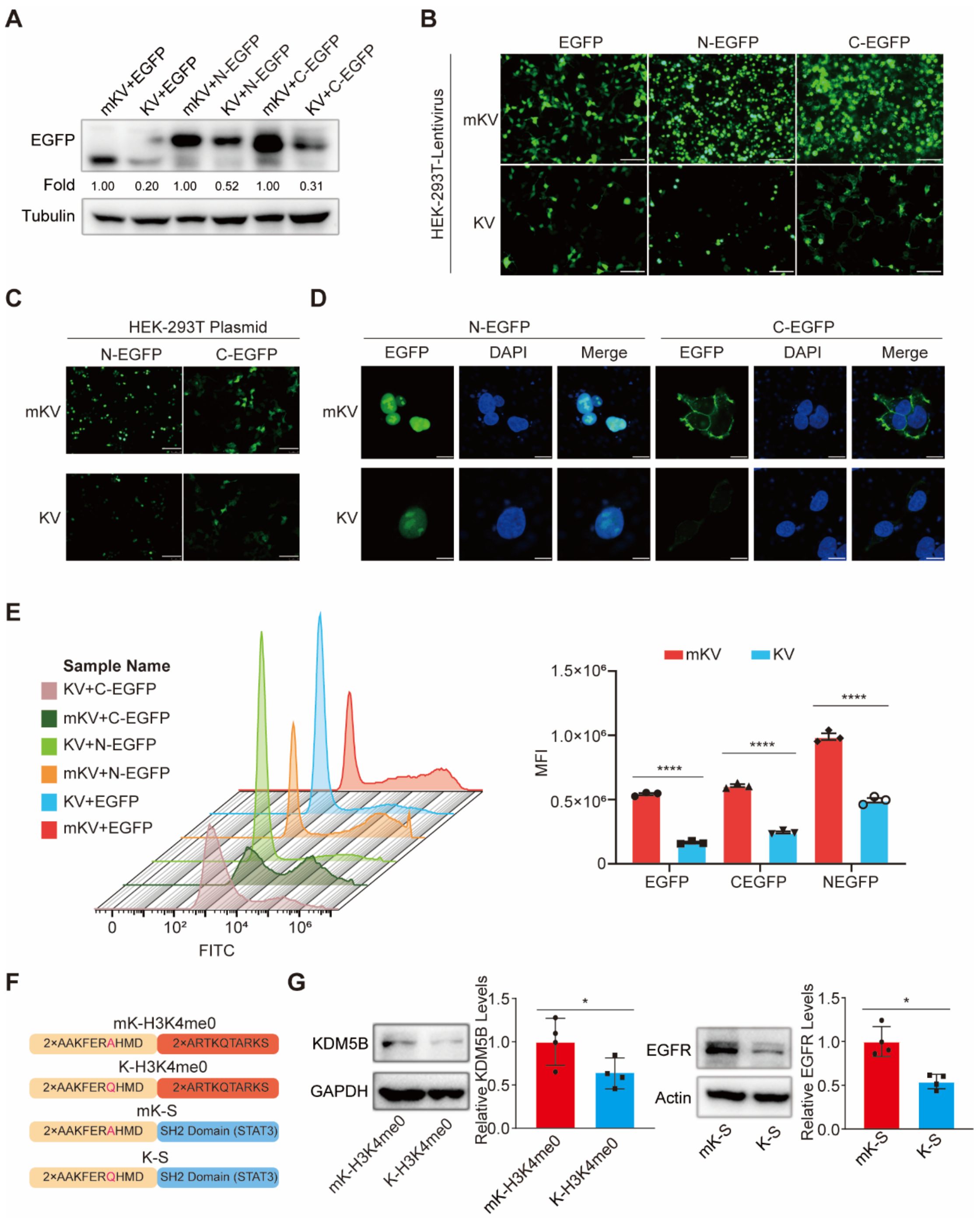

Figure S6: Knockdown of intracellular membrane proteins and nuclear proteins. (A) Western blotting showed the degradation efficiency of EGFP, NLS-EGFP (NEGFP), and CAAX-EGFP (CEGFP) in KV compared to mKV. (B) 293T cells stably overexpressing mKV or KV, followed by overexpression of EGFP, NEGFP, and CEGFP, respectively, were assessed for green fluorescence distribution and intensity with the aid of fluorescence microscopy. The scale bar indicates 100 μm. (C) The co-expression system was utilized to transiently express EGFP with mKV or KV simultaneously in 293T cells. The scale bar indicates 100 μm. And NEGFP and CEGFP were captured by confocal microscopy (D) after co-expression with mKV or KV to visualize fluorescence distribution and intensity difference. The scale bar indicates 10 μm. Green indicates

where NEGFP or CEGFP was distributed and blue indicates where the nucleus was located. And their overall EGFP levels and changes were assessed by flow cytometry (E). (F) Construction of K-H3K4 (1-10 aa) (K-H3K4me0), K-SH2 Domain (STAT3) (K-S), and corresponding control sequence. (G) Immunoblotting showed that after transfection of K-H3K4me0, K-S, and corresponding control sequence in 293T cells, the expression of KDM5B in the nucleus and EGFR on the membrane could be knocked down accordingly, and the decreased ratio of EGFR (45.8%) was slightly higher than that of KDM5B (40.6%).

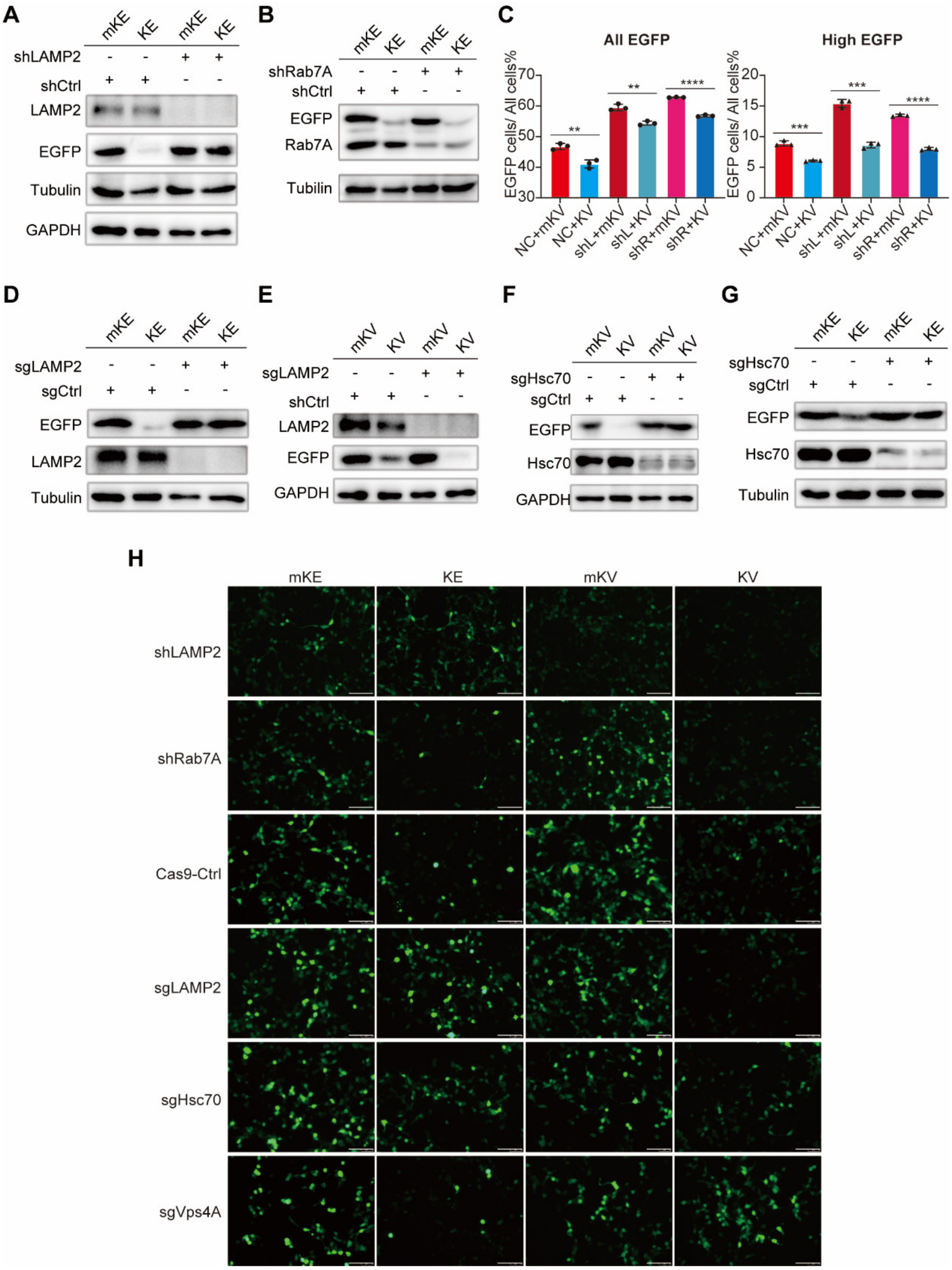

Figure S7: The degradation of EGFP by eMIATAC required Vps4A and was independent of LAMP2. (A) Western blotting showed that 293T cells with stable expression of shLAMP2 could significantly reverse the degradation of KE. (B) Western blot revealed that 293T cells with stable expression of shRabA could not significantly reverse the degradation of KE, (C) Flow cytometry was used to detect the green fluorescence expression of EGFP in 293T cells stably expressing shLAMP2 or shRab7A after co-transfection with mKV or KV. After the stable expression of sgLAMP2 and sgHsc70 in 293T cells was detected by western blotting, knockout of

LAMP2 could significantly restore the expression level of KE to similar to that of mKE (D), but still can't rescue the KV induced degradation of EGFP (E), and (F) the ability of KV to target EGFP degradation after knockout of Hsc70 was also significantly limited. (G) Immunoblot analysis showed that the degradation of KE in cells with significantly decreased Hsc70 levels would be significantly prevented. Fluorescent microscope (H) was applied to detect the effect of knockout of these proteins on KE protein degradation and the efficacy of KV targeted EGFP degradation. The scale bar indicates 100 μm.

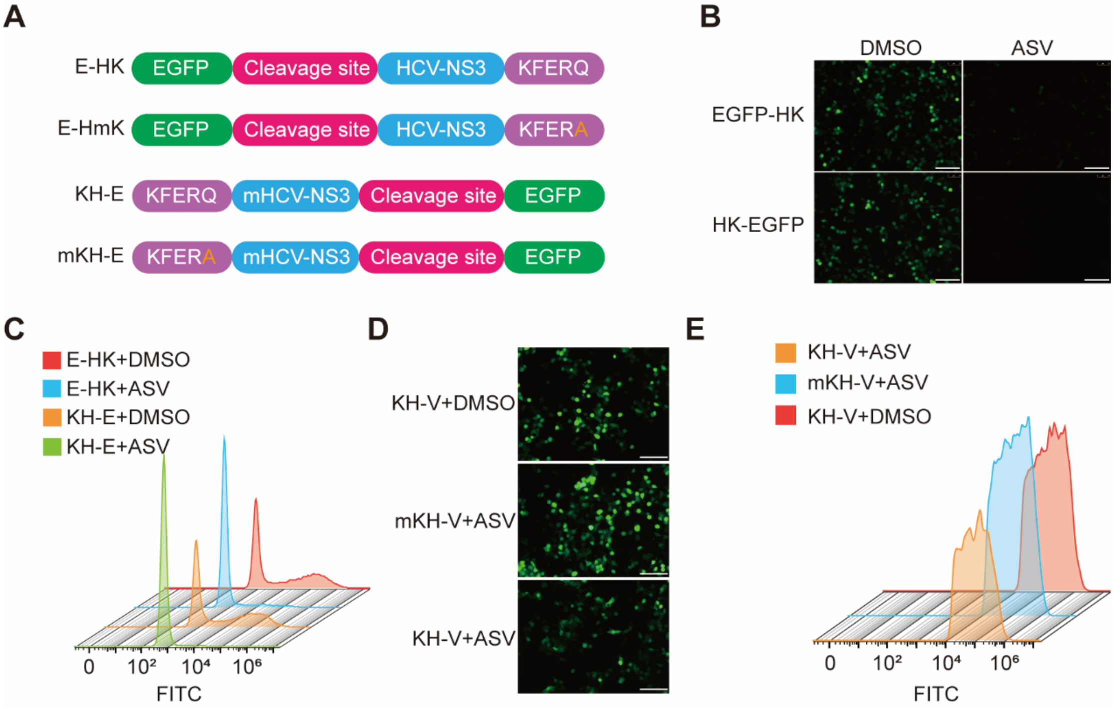

Figure S8: HCV-NS3/4A element was successfully regulated by ASV after inclusion in eMIATAC. (A) constructed E-HK, E-HmK, KH-E, and mKH-E. (B) Fluorescence photographs and (C) flow cytometry showed that E-HK and KH-E, could degrade significantly with the ASV, and the KFERQ motif could achieve better degradation power at the N terminal of HCV NS3. The scale bar indicates 100 μm. (D) Fluorescence photographs and flow cytometry (E) showed that KH-V and the action of ASV significantly regulated the degradation of EGFP. The scale bar indicates 100 μm.

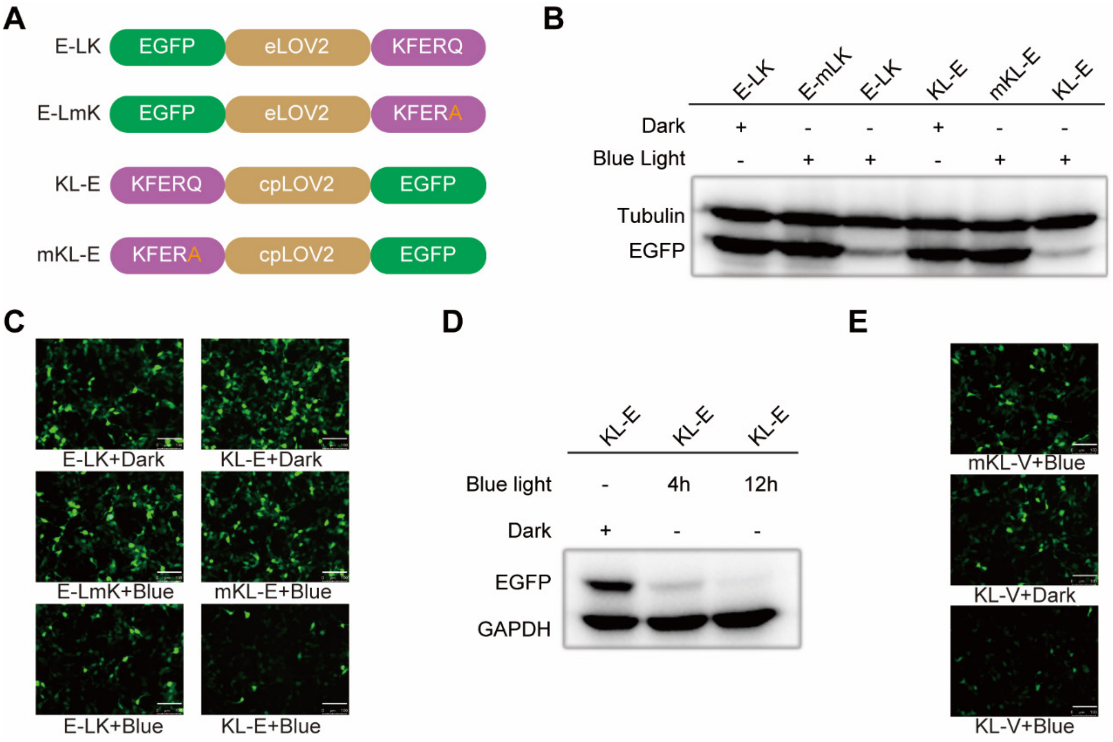

Figure S9: cpLOV2 element was successfully regulated by blue-light after inclusion in eMIATAC. (A) E-LK, E-LmK, KL-E, and mKL-E sequence schematic. (B) Immunoblot showed differences in degradation efficiency of N-terminus targeting KFERQ motif located at LOV2 or cpLOV2. (C) Fluorescence microscopy was used to determine the green fluorescence intensity and level of E-LK, E-LmK, KL-E, and mKL-E under dark or blue light (470nm, 4mW/cm^2) conditions. The scale bar indicates 100 µm. (D) Immunoblotting showed the time-dependent degradation of KL-E after exposure to blue-light (470nm, 4mW/cm^2). (E) The regulation effect of mK/KL-V on EGFP under dark and blue light (470nm, 4mW/cm^2) was determined by fluorescence microscopy. The scale bar indicates 100 µm.

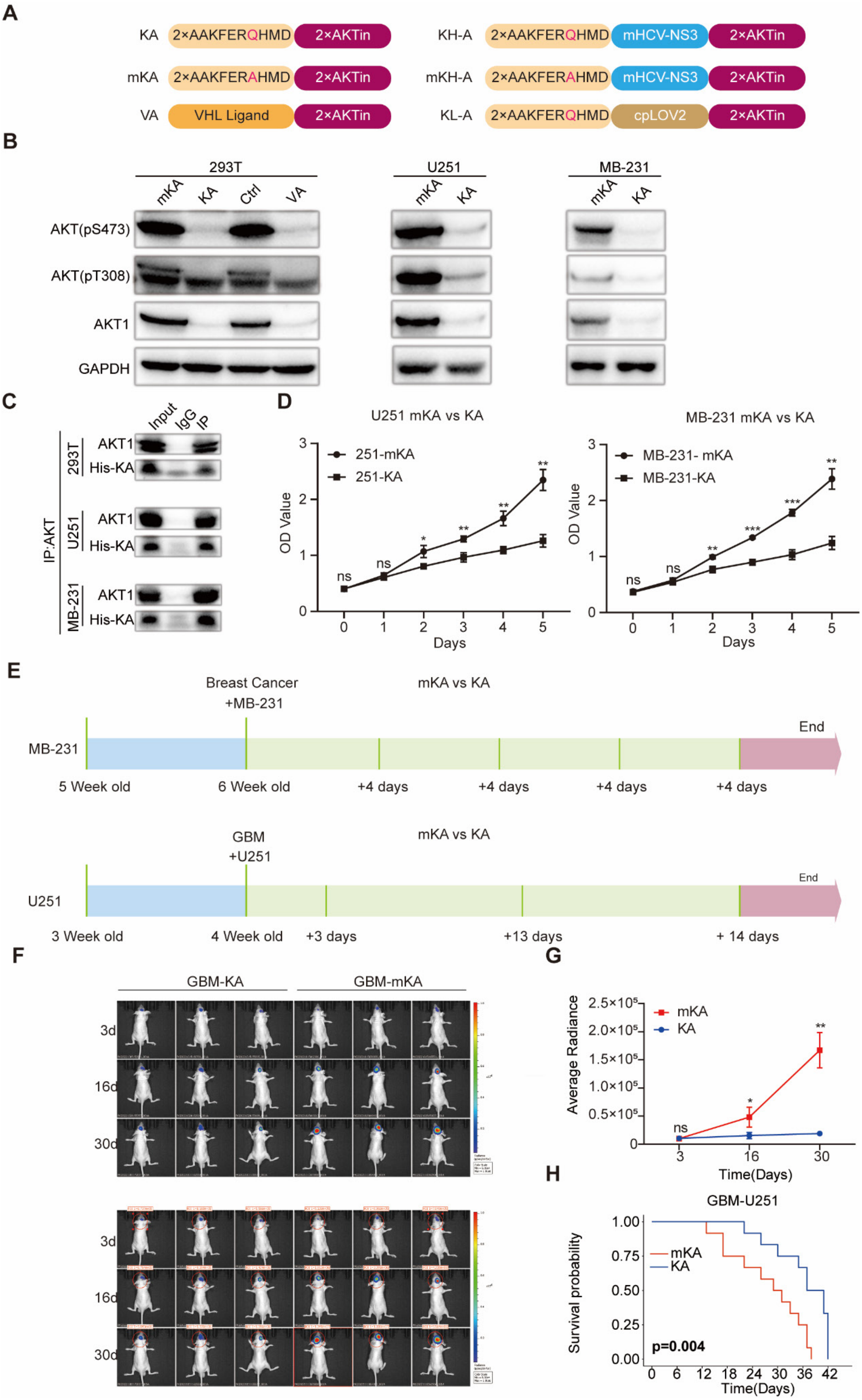

A
KA 2×AAKFERQHMD 2×AKTin
mKA 2×AAKFERAHMD 2×AKTin
VA VHL Ligand 2×AKTin
KH-A 2×AAKFERQHMD mHCV-NS3 2×AKTin
mKH-A 2×AAKFERAHMD mHCV-NS3 2×AKTin
KL-A 2×AAKFERQHMD cpLOV2 2×AKTin
B
293T
mKA KA Ctrl VA
U251
mKA KA
MB-231
mKA KA
AKT(pS473)
AKT(pT308)
AKT1
GAPDH
C
Input IgG IP
IP:AKT
293T
U251
MB-231
AKT1
His-KA
D
U251 mKA vs KA
251-mKA
251-KA
MB-231 mKA vs KA
MB-231- mKA
MB-231-KA
OD Value
Days
ns
*
**
***
E
Breast Cancer
+MB-231
mKA vs KA
End
MB-231
5 Week old
6 Week old
+4 days
GBM
+U251
U251
3 Week old
4 Week old
+3 days
+13 days
+ 14 days
F
GBM-KA
GBM-mKA
3d
16d
30d
G
Average Radiance
mKA
KA
Time(Days)
H
GBM-U251
Survival probability
mKA
KA
p=0.004
Time(Days)

Figure S10: Application of eMIATAC in degradation of endogenous AKT1 *in vitro*. (A) Designed and constructed KA, mKA, VA, KH-A, mKH-A and KL-A. (B) Western blotting showed that after mKA and KA were stably expressed in 293T, U251, and MB-231 cells, KA could steadily reduce the levels of endogenous AKT1 and pAKT, and the knockdown effect in 293T cells was similar to VA. (C) His-KA was stably overexpressed in 293T, U251, and MB-231 cells, and cell lysates were taken for co-immunoprecipitation. The results supported the strong interaction between His-KA and AKT1. (D) Compared with the control group, the proliferation of U251 and MB-231 cells was significantly inhibited after stable expression of KA by CCK8 assay. (E) Subcutaneous BRCA model (MB-231) and intracranial glioma model (U251) were constructed in 4-week-old nude mice, as well as a follow-up treatment flow chart. (F) The results of bioluminescence imaging showed that the glioma tissue of the KA group had a lower luminescence intensity than that of the mKA group, that is, a smaller tumor volume. (G) After taking pictures at appropriate time points, we conducted statistics and found that the difference in tumor size between the mKA side and the KA side continued to increase. The difference in glioma survival between mKA and KA groups was shown in (H).

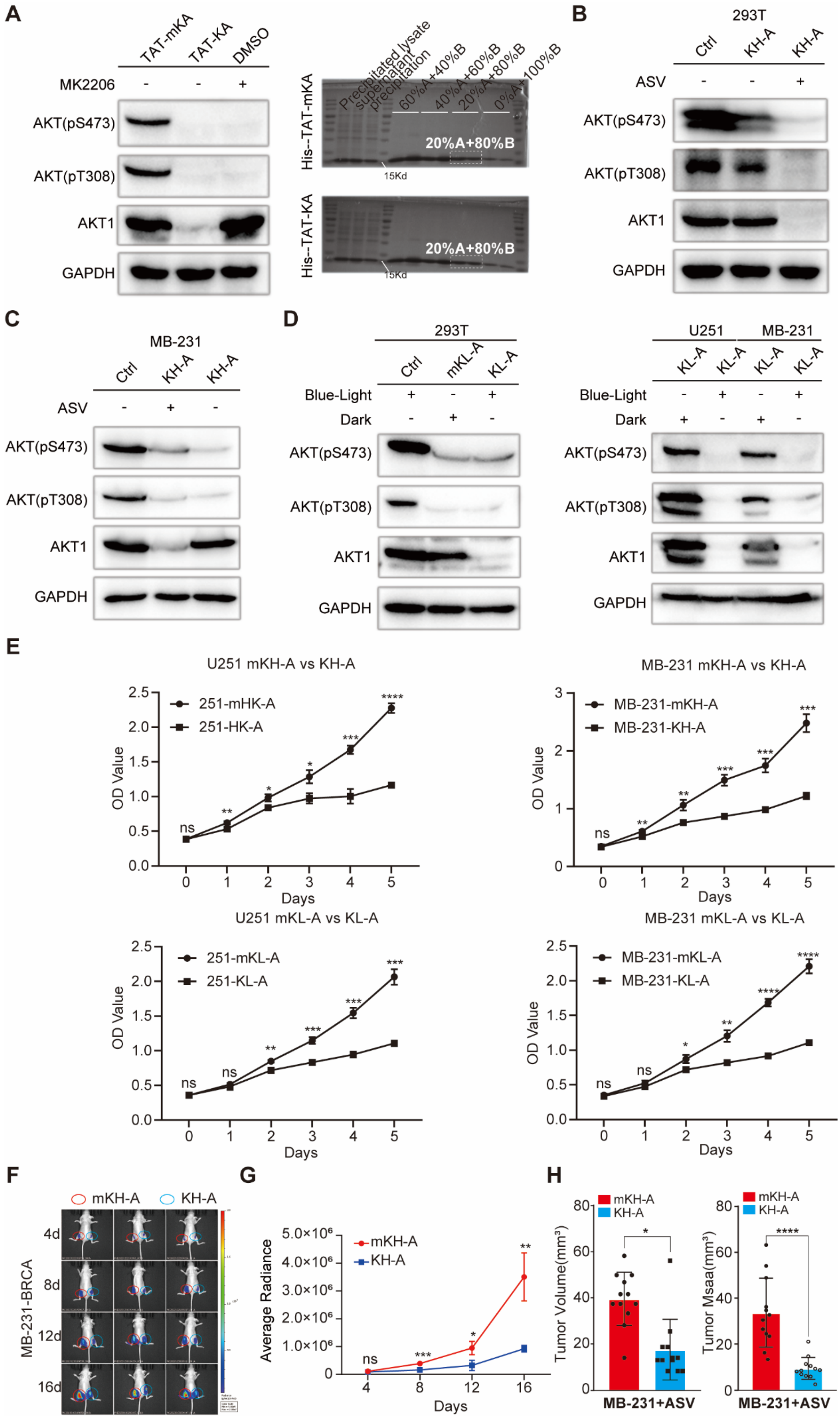
A
TAT-mKA
TAT-KA
DMSO
MK2206
AKT(pS473)
AKT(pT308)
AKT1
GAPDH
Precipitated lysate
supernatant
precipitation
60%A+40%B
40%A+60%B
20%A+80%B
0%A+100%B
His--TAT-mKA
20%A+80%B
15Kd
His--TAT-KA
20%A+80%B
15Kd
B
293T
Ctrl
KH-A
KH-A
ASV
AKT(pS473)
AKT(pT308)
AKT1
GAPDH
C
MB-231
Ctrl
KH-A
KH-A
ASV
AKT(pS473)
AKT(pT308)
AKT1
GAPDH
D
293T
Ctrl
mKL-A
KL-A
Blue-Light
Dark
AKT(pS473)
AKT(pT308)
AKT1
GAPDH
U251
MB-231
KL-A
Blue-Light
Dark
AKT(pS473)
AKT(pT308)
AKT1
GAPDH
E
U251 mKH-A vs KH-A
251-mHK-A
251-HK-A
OD Value
Days
MB-231 mKH-A vs KH-A
MB-231-mKH-A
MB-231-KH-A
OD Value
Days
U251 mKL-A vs KL-A
251-mKL-A
251-KL-A
OD Value
Days
MB-231 mKL-A vs KL-A
MB-231-mKL-A
MB-231-KL-A
OD Value
Days
F
mKH-A
KH-A
MB-231-BRCA
4d
8d
12d
16d
G
Average Radiance
mKH-A
KH-A
Days
H
mKH-A
KH-A
Tumor Volume(mm³)
MB-231+ASV
mKH-A
KH-A
Tumor Msaa(mm³)
MB-231+ASV

Figure S11: The eMIATAC degrades endogenous AKT1 and pAKT in vivo. (A) Left, western blotting showed that His-TAT-KA inhibited both AKT1 and pAKT levels in 293T cells, unlike MK2206, which could only significantly reduce pAKT levels. AKTin could effectively inhibit the activity of AKT, but could not significantly induce the degradation of total AKT1. Right, Coomath blue staining showed that both His-TAT-mKA and His-TAT-KA could be purified effectively. In 293T (B) and MB-231 (C) cells, compared with the control group, DMSO treatment could only inhibit the level of pAKT in the stably expressed HK-A cells, but the level of AKT1 and pAKT could be significantly reduced after the addition of ASV. (D) Immunoblotting revealed that 293T cells expressing the AKT1 targeting eMIATAC system with cpLOV2 displayed lower AKT1 and pAKT levels in response to blue-light (470nm,4mW/cm^2) compared to controls, and this eMIATAC had no significant effect on total AKT1 in the absence of blue-light. (E) CCK8 assay confirmed that cell proliferation was significantly inhibited after stable expression of KH-A and KL-A in U251 and MB-231 cells compared with the control group. (F) The results of bioluminescence imaging showed that the glioma tissue of the KH-A group had a lower luminescence intensity than that of the mKH-A group under the effect of ASV, that is, a smaller tumor volume. The size of BRCA tissue was indicated by the intensity of bioluminescence. (G) After taking pictures at appropriate time points, we conducted statistics and found that the difference in tumor size between the HK-A side and the HK-A side continued to increase. (H) The constructed BRCA model mice (mHK-A vs HK-A) were surgically stripped of the subcutaneous tumor tissue 19 days after tumor formation, and the tumor volume and mass were measured respectively. It was found that the mice in the HK-A group could significantly inhibit the growth of the tumor tissue under the induction of ASV.

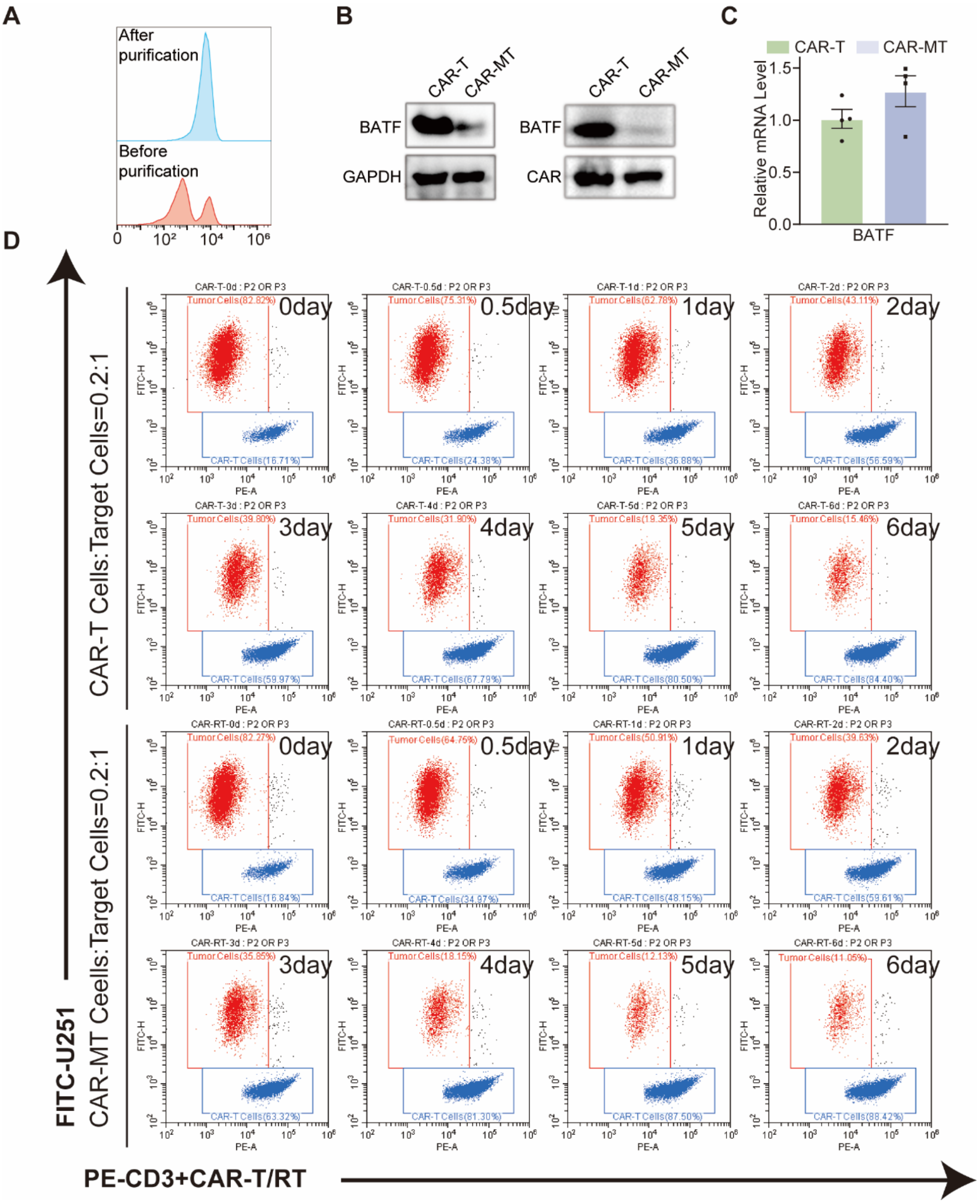

Figure S12: *In vitro* validation of CAR-MT. (A) The proportion of CD3+T cells before and after PMBC purification. The protein levels (B) and mRNA (C) of BATF in CAR-T and CAR-MT cells. (D) The remaining proportion of U251 cells at different time points after co-culturing CAR-T or CAR-MT with U251 EGFP cells at a ratio of 0.2:1 (E: T).

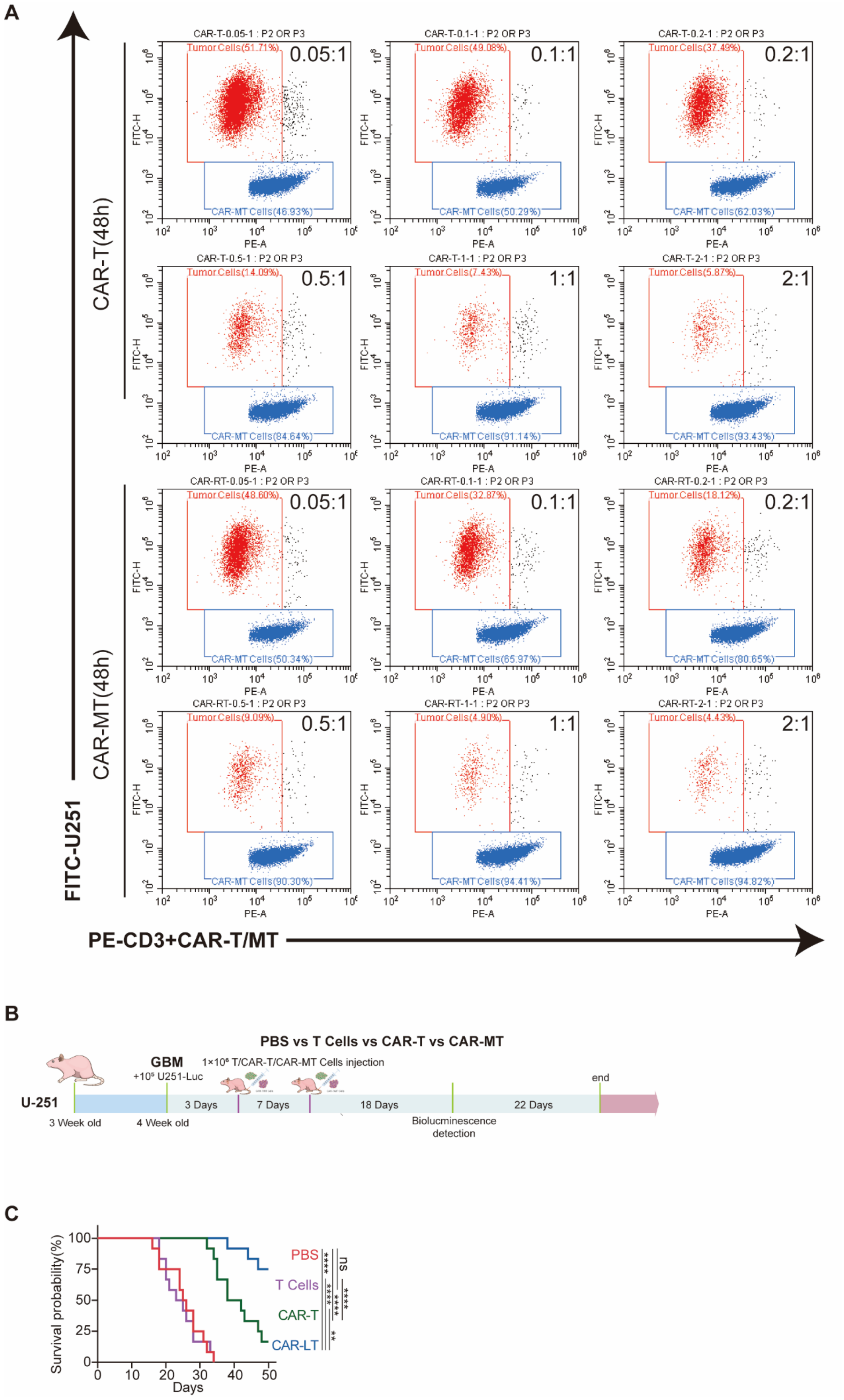
A
CAR-T(48h)
CAR-MT(48h)
FITC-U251
PE-CD3+CAR-T/MT
CAR-T-0.05-1 : P2 OR P3
Tumor Cells(51.71%)
0.05:1
CAR-MT Cells(46.93%)
CAR-T-0.1-1 : P2 OR P3
Tumor Cells(49.08%)
0.1:1
CAR-MT Cells(50.29%)
CAR-T-0.2-1 : P2 OR P3
Tumor Cells(37.49%)
0.2:1
CAR-MT Cells(62.03%)
CAR-T-0.5-1 : P2 OR P3
Tumor Cells(14.09%)
0.5:1
CAR-MT Cells(84.64%)
CAR-T-1-1 : P2 OR P3
Tumor Cells(7.43%)
1:1
CAR-MT Cells(91.14%)
CAR-T-2-1 : P2 OR P3
Tumor Cells(5.87%)
2:1
CAR-MT Cells(93.43%)
CAR-RT-0.05-1 : P2 OR P3
Tumor Cells(48.60%)
0.05:1
CAR-MT Cells(50.34%)
CAR-RT-0.1-1 : P2 OR P3
Tumor Cells(32.87%)
0.1:1
CAR-MT Cells(65.97%)
CAR-RT-0.2-1 : P2 OR P3
Tumor Cells(18.12%)
0.2:1
CAR-MT Cells(80.65%)
CAR-RT-0.5-1 : P2 OR P3
Tumor Cells(9.09%)
0.5:1
CAR-MT Cells(90.30%)
CAR-RT-1-1 : P2 OR P3
Tumor Cells(4.90%)
1:1
CAR-MT Cells(94.41%)
CAR-RT-2-1 : P2 OR P3
Tumor Cells(4.43%)
2:1
CAR-MT Cells(94.82%)
FITC-H
PE-A
B
PBS vs T Cells vs CAR-T vs CAR-MT
U-251
GBM
+10⁵ U251-Luc
1×10⁶ T/CAR-T/CAR-MT Cells injection
3 Week old
4 Week old
3 Days
7 Days
18 Days
22 Days
end
Biolucminescence detection
C
Survival probability(%)
Days
PBS
T Cells
CAR-T
CAR-LT
ns
****
**

Figure S13: (A)The remaining proportion of U251 cells after co-culturing CAR-T or CAR-MT with U251 EGFP cells at different E:T ratios. (B) Monitoring the survival status of mice after T cell injection.

Table S1: The shRNA and sgRNA targeting sequences.

Gene	TargetSeq1	TargetSeq2 (data not shown)	TargetSeq3 (data not shown)
shRab7A	GGAAGACATCACTCATGAACC	GGCTAGTCACAATGCAGATAT	GCTGTACAACGAATTTCCTGA
shLAMP2	GCTCGTTCTGGTCTGCCTAGT	GCTCTACTTAGACTCAATAGC	GCAATAGTTTATCAACTTTGG
sgLAMP2	ATAGCAGTGCAGTTCGGACC	CTTGGTAAAATTCGCAATCC	TCCGAACTGCACTGCTATTT
sgVps4A	CACGTCGTTCCACCGTATGT	ACGTCGTTCCACCGTATGTT	CAACATACGGTGGAACGACG
sgHsc70	ACGGACACTGAACGGTTGAT	ACAGATGCCAAACGTCTGAT	TTCAAATCGGGCACGGGTAA
